# The impact of management on the fecal microbiome of endangered greater sage-grouse (*Centrocercus urophasianus*) in a zoo-based conservation program

**DOI:** 10.1093/conphys/coae052

**Published:** 2024-08-07

**Authors:** Emma Vaasjo, Mason R Stothart, Sandra R Black, Jocelyn Poissant, Douglas P Whiteside

**Affiliations:** Faculty of Veterinary Medicine, University of Calgary, 3280 Hospital Dr NW, Calgary, AB T2N 4Z6, Canada; Animal Health Department, Wilder Institute/Calgary Zoo, 1300 Zoo Rd NE, Calgary, AB T2E 7V6, Canada; Faculty of Veterinary Medicine, University of Calgary, 3280 Hospital Dr NW, Calgary, AB T2N 4Z6, Canada; Animal Health Department, Wilder Institute/Calgary Zoo, 1300 Zoo Rd NE, Calgary, AB T2E 7V6, Canada; Faculty of Veterinary Medicine, University of Calgary, 3280 Hospital Dr NW, Calgary, AB T2N 4Z6, Canada; Faculty of Veterinary Medicine, University of Calgary, 3280 Hospital Dr NW, Calgary, AB T2N 4Z6, Canada; Animal Health Department, Wilder Institute/Calgary Zoo, 1300 Zoo Rd NE, Calgary, AB T2E 7V6, Canada

**Keywords:** Centrocercus urophasianus, conservation, conservation breeding, gastrointestinal, greater sage-grouse, managed care, microbiome

## Abstract

Greater sage-grouse (*Centrocercus urophasianus*) are a critically endangered species in Canada with fewer than 140 individuals remaining on native habitats in southern Alberta and Saskatchewan. In 2014, the Wilder Institute/Calgary Zoo initiated North America’s only zoo-based conservation breeding program for this species to bolster declining wild populations through conservation reintroductions. Within the managed population of sage-grouse, morbidity and mortality have primarily been associated with intestinal bacterial infections. As a preliminary study to assess the gastrointestinal health of this species in managed care, the fecal bacterial microbiome of adult and juvenile captive sage-grouse was characterized with 16S rRNA sequencing. The composition of the microbiome at the phylum level in greater sage-grouse is consistent with previous studies of the avian microbiome, with *Bacillota* as the most abundant phyla, and *Actinomycetota*, *Bacteroidota* and *Pseudomonadota* also being highly abundant. Antibiotic use and sex did not have a significant impact on the diversity or composition of the microbiome, but the management of juvenile sage-grouse did influence the development of the microbiome. Juveniles that were raised outdoors under maternal care developed a microbiome much more similar to adults when compared to chicks that were incubated and hand-raised. The local environment and parental care appear to be important factors influencing the diversity and composition of the gastrointestinal microbiome in this species.

## Teaser

This study characterizes the bacterial fecal microbiome of a managed flock of endangered greater sage-grouse, an avian dietary specialist. The effects of sex, age, antibiotic-use, and incubation strategies were considered. Incubation and hand-rearing was shown to have a significant impact on delaying the development of the microbiome compared to hen-rearing.

## Introduction

The effects of managed care on the gastrointestinal microbiome have been studied in various species, but avian studies are relatively sparse (Sun *et al.*, 2022). Across terrestrial vertebrate taxa, studies describe a decrease in diversity and/or a significant difference in the composition of microbial communities in managed individuals compared to their wild counterparts (Eigeland *et al.*, 2012; Clayton *et al.*, 2016; Delport *et al.*, 2016; Metcalf *et al.*, 2017; Jia *et al.*, 2018; Guo *et al.*, 2019; Hernández-Gómez *et al.*, 2019). In occasional cases, an increase in microbial diversity in managed animals has been observed, while studies on both cutaneous and intestinal microbiota in several species show no difference between wild and managed individuals (Alfano *et al.*, 2015; Flechas *et al.*, 2017; McKenzie *et al.*, 2017). These findings highlight the need for individual studies on species under human care, as each species appears to differ in their ability to develop or conserve their wild microbiome in a managed environment.

Antibiotics are commonly used to treat bacterial infections causing disease in managed populations; though potentially lifesaving, their use can lead to dysbiosis, other disease states, or the extirpation of beneficial microbiota (Petersen and Round, 2014; West *et al.*, 2019). While there are many studies on disruptions to the microbiome after antibiotic use in humans, similar studies on threatened and endangered species are limited (Vlčková *et al.*, 2016; Dahlhausen *et al.*, 2018; West *et al.*, 2019). The consequences of variation in microbial communities on host health may not be apparent until post-release if microbial species critical to survival in the wild have been lost in captivity (West *et al.*, 2019).

The greater sage-grouse (*Centrocercus urophasianus*, hereafter sage-grouse), the largest grouse species in North America and a flagship species for sagebrush habitat, is critically endangered in Canada. Listed on the Species at Risk Act since 1998, sage-grouse have been lost from 90% of their historic Canadian range, with small, isolated populations remaining in southern Alberta and Saskatchewan. Extant populations have suffered significant population declines in the last three decades (Connelly *et al.*, 2000; Aldridge and Brigham, 2003; Schroeder *et al.*, 2004). In winter, sage-grouse are specialists, consuming a largely monospecific diet of chemically toxin-rich sagebrush. Wild sage-grouse have enlarged ceca with important microbial populations that aid in the digestion and detoxification of sage (Wienemann *et al.*, 2011; Kohl *et al.*, 2016).

In 2014, the Wilder Institute/Calgary Zoo initiated North America’s only zoo-based conservation breeding program for this species to bolster declining wild populations through conservation reintroductions. The first releases of young of the year birds to wild habitats occurred in the autumn of 2018 and have continued annually. Unfortunately, survival rates in these juveniles post-release have been extremely low at less than 2% (Black *et al.*, 2019). Within the captive population, the largest proportion of morbidity and mortality (approximately 60–80% per year) have been associated with diseases arising from intestinal bacterial infections, seen especially in the neonatal and juvenile population (Black *et al.*, 2019; Pastor *et al.*, 2021). A lack of diversity and/or richness of a more wild-type microbial community may be negatively affecting the health of sage-grouse under human care and may also impact their ability to survive once released back into native habitats.

The purpose of this research was to characterize the microbiome of sage-grouse under managed care in a zoo-based conservation program and describe community changes of the microbiome in relation to sex, growth, neonatal management strategies and antibiotic use. Findings will aid in the management of sage-grouse under human care and help to determine if altered microbiome diversity possibly contributes to low survival post-release.

## Materials and methods

### Sample population

The flock at the Wilder Institute/Calgary Zoo was founded by the collection of eggs from wild sage-grouse hens in Montana, Saskatchewan and Alberta in 2014 (Heinrichs *et al.,* 2019). At the time of sample collection, the number of breeding adults was maintained between 50 and 60 individuals, with the goal of hatching 200–250 chicks annually. All birds were offered the same diet, a commercial formulated dry pellet; game-bird starter as chicks (Mazuri) or an in-house developed pheasant breeder pellet as adults (Country Junction Feeds), with the addition of lettuce, chopped greens, cucumber, peas, edamame, mealworms and crickets. Dried sage was also offered in bundles and sage plants were used when available. From 2018 to 2021, a diet transition over a period of 4–5 weeks was used for juveniles leading up to their release to native wild habitats. During this transition, pelleted food was slowly decreased with a corresponding increase in vegetables and legumes and the introduction of fresh sage.

### Sample collection

This research was approved by the Calgary Zoo Welfare, Ethics and Research Committee (CZWERC 2020-02) and by the University of Calgary Veterinary Sciences Animal Care Committee (AC20-0037).

The study group consisted of 10 healthy adult females and 10 healthy adult males, with a representative number of adults from each age group (age ranged from 1 to 5 years). Males were chosen randomly, while females housed alone during chick rearing were preferentially selected to decrease disturbance to nesting and brood rearing. If adults died during the course of the study, they were replaced with birds of the same sex and a similar age to maintain a consistent sample size throughout the sampling period.

Once hatched, one cohort of three to five incubated chicks was recruited into the study each week for 4 weeks. Six broods of hen-raised chicks were also selected and followed from hatch. Following mortalities in the incubated group and random selection from the hen-raised group, there were 15 incubated and hand-reared and 15 hen-raised chicks that were followed for the length of the study, with additional samples from other chicks collected opportunistically.

Sample collection commenced in June 2020. At least one fresh sample was collected monthly from adults, apart from September and October 2020 while the flock was undergoing intensive treatment and testing due to an avian malaria outbreak. If animal care staff observed an individual defecate, or the adult was placed in a crate for any reason (weight checks, transport between pens, or veterinary exam), a sample was collected. Occasionally, adults in group housing were administered a food safe non-toxic glitter in superworms or peas and a fecal sample was able to be identified from that specific individual 3–4 hours later.

Weekly samples were collected from both hen-raised (group samples or individual samples while being weighed) and artificially incubated chicks until they were moved into the larger flight pen. Three groups of juveniles were moved to soft release pens on wild native habitat during the fall of 2020 (two in Alberta and one in Saskatchewan) and multiple group samples were collected from these soft release pens at least one week apart.

Information on date, time and the substrate from which the sample was collected, and antibiotic exposure were recorded for each sample. Juveniles were treated as antibiotic-exposed if they had any history of antibiotic administration. As every adult bird within the breeding flock had previously been treated with antibiotics sometime during their time in managed care, antibiotic exposure for each individual sample was defined based on administration within the last 6 months, and the number of days since their last antibiotic administration was also recorded. Juvenile samples were also placed into a binary variable for management type (hen-raised versus incubated and hand-raised) and grouped into age cohorts to facilitate some analyses (see Table S1 for collection data for all samples used for analysis). Samples were immediately placed in a −20°C freezer and later moved to a −80°C freezer until analysis.

### DNA extraction, PCR amplification and sequencing

A total of 311 samples (0.25 g each, or entire sample if < 0.25 g) were placed in Powerbead Pro Plates (Qiagen Sciences) with one empty well in each plate (negative control) and shipped overnight on dry ice to Genome Quebec (Montreal, Canada).

Plates were thawed for 1–2 hours, then total DNA was extracted with the standard protocol for DNeasy 96 PowerSoil Pro QIAcube HT kit (Qiagen Sciences) using the QIAcube HT extraction robot. TissueLyserII (Qiagen Sciences) was used for bead beating. One plate was run per day. Final DNA extracts were eluted in 100 μl of TopElute buffer and stored at −20°C until library preparation.

The fourth variable region (V4) of the 16S rRNA gene (252–254 bp) was amplified using 5 μl of DNA extract and primers 515FB and 806RB as outlined by the Earth Microbiome Project (Caporaso *et al.*, 2012; Thompson *et al.*, 2017; Drovetski *et al.*, 2019). Only 35% of samples were successfully amplified based on DNA quantification and gel electrophoresis, likely due to low initial DNA concentrations after extraction (<1 ng/μl, quantified with Quant-iT PicoGreen dsDNA Assay Kit, Life Technologies). Sequencing libraries were prepared by pooling a standardized amount of PCR products from all samples (including those that were not successfully amplified). SparQ PureMag Beads (Quantabio) were used to clean libraries and fragment sizes were verified using a LabChip GX (PerkinElmer) instrument prior to sequencing on an Illumina MiSeq platform (v2 chemistry, 250-bp paired-end reads, 500 cycles) at Genome Quebec.

Sequencing data from MiSeq platform was de-multiplexed and converted to Fastq format using Illumina’s bcl2fastq software. Cutadapt v1.16 was used to remove primers and for initial quality trimming with minimum quality score of 20 (Martin, 2011). Trimmed reads were then processed by using the DADA2 (v 1.22.0) pipeline to clean, deduplicate and merge forward and reverse reads to generate an amplicon sequence variant (ASV) table (Callahan *et al.*, 2016). Taxonomy was assigned to the representative sequences using the RDP classifier (Wang *et al.*, 2007; Lan *et al.*, 2012) and the SILVA v138.1 database (Quast *et al.*, 2013; Yilmaz *et al.*, 2014) using an 80% confidence threshold. Only ASVs identified as bacterial were retained. Phylogenetic distances among ASVs were estimated using a relaxed neighbour-joining method via mothur implementation of clearcut (Sheneman *et al.*, 2006; Kozich *et al.*, 2013). An unexpectantly large number of paired reads were obtained from two out of four negative controls (10 135 and 14 345), which may have been caused by spillover from neighbouring wells during the DNA extraction process.

Although over 300 samples were extracted and sequenced, 220 samples (including samples from both chicks and adults) had less than 8000 reads and were excluded from analyses. Rarefaction curves demonstrated that a sampling depth of 8000 reads was sufficient to estimate diversity (Fig. S1). Most samples were unusable likely due to issues with low yields from DNA extraction, a known issue for avian feces when using commercially available kits (Eriksson *et al.*, 2017; Edwards *et al.*, 2023). The final sample size used for analysis was *n* = 35 from 20 adults and *n* = 56 from juveniles (44 samples from 23 chicks, 6 group samples from 4 hen-raised broods and 6 group samples from soft release pens). After the quality control methods described above were completed, the number of sequences obtained from single samples used for analysis ranged from 8112 to 165 628 with a median of 41 239. Unless otherwise specified, samples were normalized by rarefying read counts to the lowest observed sequencing depth (8112 reads) prior to analysis.

### Statistical analyses

Statistical analysis was performed using R v4.2.1 (R Core Team, 2021) and various packages including phyloseq v1.40.0 (McMurdie and Holmes, 2013) for data handling, vegan v2.6-4 (Dixon, 2003) and lmerTest v.3.1–3 (Kuznetsova *et al.*, 2017) for statistical analysis and ggplot2 v3.3.6 (Wickham *et al.*, 2016) for visualization. The R script used for analyses can be found in the supplementary material.

Alpha diversity was measured by calculating the observed number of ASVs per sample as well as Simpson and Shannon indexes (Simpson, 1949; Spellerberg and Fedor, 2003). Linear mixed effects models were used to model alpha diversity estimates as a response to fixed effects for sex, antibiotic use and sample collection substrate/location among adult samples, or management type (hen- vs. hand-raised), age (as a continuous variable) and antibiotic use among juvenile sample tests. Additional models were used to test for differences in alpha diversity between adult and juvenile samples that included age-class (adult vs juvenile) or age cohort as a covariate. Age cohorts separated juvenile samples into five groups: 1–2 weeks, 3–4 weeks, 5–6 weeks, 7–12 weeks and > 12 weeks. Individual bird identity was included as a random intercept in all models to account for multiple samples collected from the same individual. Samples from the oldest juveniles in soft release pens were excluded from alpha diversity assessments based on management and antibiotic use as only group samples could be collected. Group samples consisted of a mixture of several different fresh fecal samples collected from the environment, without confirmation on which birds within a specific pen produced the collected fecal material.

Aitchison distances from non-rarefied count tables were used as one measure of beta diversity, alongside estimates of weighted and unweighted UniFrac distances calculated from rarefied datasets. Permutational multivariate analysis of variance (PERMANOVA; 999 permutations, Anderson, 2017, Kelly *et al.*, 2015) were used to determine the marginal effects of age class (adult or juvenile), antibiotic use (both as a binary variable and days since last antibiotic dose as a continuous variable for adults), age, sex, management style (hen vs. hand raised) and sample collection location on phylogeny-weighted/unweighted measures of microbiome beta-diversity. Additional tests for multivariate homogeneity of groups dispersions using the function betadisper() from the R package ‘vegan’ were applied to fixed effects found in PERMANOVA analyses to be significantly associated with measures of beta diversity. Post hoc Tukey honest significant difference tests were then performed to determine the direction of differences in beta dispersion among pairwise comparisons.

To more directly compare adult microbiomes to juvenile microbiomes, juvenile samples were categorized into five age cohorts as described above. Cohort 5 chicks (>12 weeks) had been moved to soft release pens closer to release sites and had undergone a diet transition to reduce pelleted feed. As group samples were included in these comparisons, individual-linked variables on management and antibiotic use could not be included.

When needed, the R package pairwiseAdonis v.0.4 was used to make pairwise comparisons (Martinez Arbizu, 2017) and *P*-values were corrected for multiple comparisons using the Holm–Bonferroni method (Holm, 1979).

Differential abundance analysis between adults based on antibiotic use, juveniles based on management type and juveniles and adults were assessed at the family level using a non-rarefied count table and the function ANCOMBC from the eponymous package, v.1.6.3 (Lin and Peddada, 2020; Nearing *et al.*, 2022). As above, Holm–Bonferroni corrections were applied to account for multiple comparisons (Holm, 1979).

## Results

### Microbiome composition

**Figure 1:**
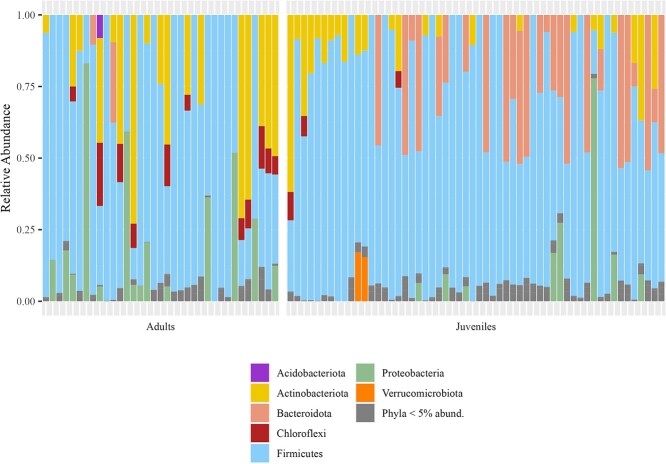
Bar plot of the relative abundance of the seven most abundant bacterial phyla sequenced from the sage-grouse fecal microbiome. Phyla whose median relative abundance were less than five percent are combined and represented in grey. Each column represents an individual sample, separated by sample type.

A total of 11 bacterial phyla were present between all sequenced samples (Fig. 1). *Bacillota* was the most abundant phyla in both adults (64.6% ± 5% *SE*) and juveniles (69.9% ± 3.1% *SE*). *Actinomycetota* was the second most abundant phylum in adults (18.1% ± 3.9% *SE*) but was less abundant in juveniles (8% ± 1.5% *SE)*. The second most abundant phylum in juvenile samples was *Bacteroidota* (16.2% ± 2.7% *SE*), which was found in lower abundance in adult samples (1.5% ± 0.9% *SE*). *Pseudomonadota* was also abundant in both sample types (11.2% ± 3.9% *SE* in adults and 4.1% ± 1.5% *SE* in juveniles).

### Characterization of the adult microbiome

No significant differences in alpha diversity were noted based on sex, antibiotic use or sample location when considering Observed, Simpson or Shannon measures of alpha diversity (Table S2). Similarly, no significant differences in beta diversity were noted on the basis of sex (*n* = 15 males, *n* = 20 females), or location of sample collection (Table S3). Beta diversity was, however, found to vary marginally based on the binary variable for antibiotic use in the past six months for both Aitchison (*P* = 0.04) and unweighted UniFrac distances (*P* = 0.04, Table S3), as well as the continuous variable for number of days since the last antibiotic treatment for both Aitchison (*P* = 0.03) and weighted UniFrac distances (*P* = 0.04, Table S3). Despite these measured differences, there were no bacterial families that significantly differed in abundance based on the binary variable for antibiotic use.

### Characterization of the juvenile microbiome

**Figure 2:**
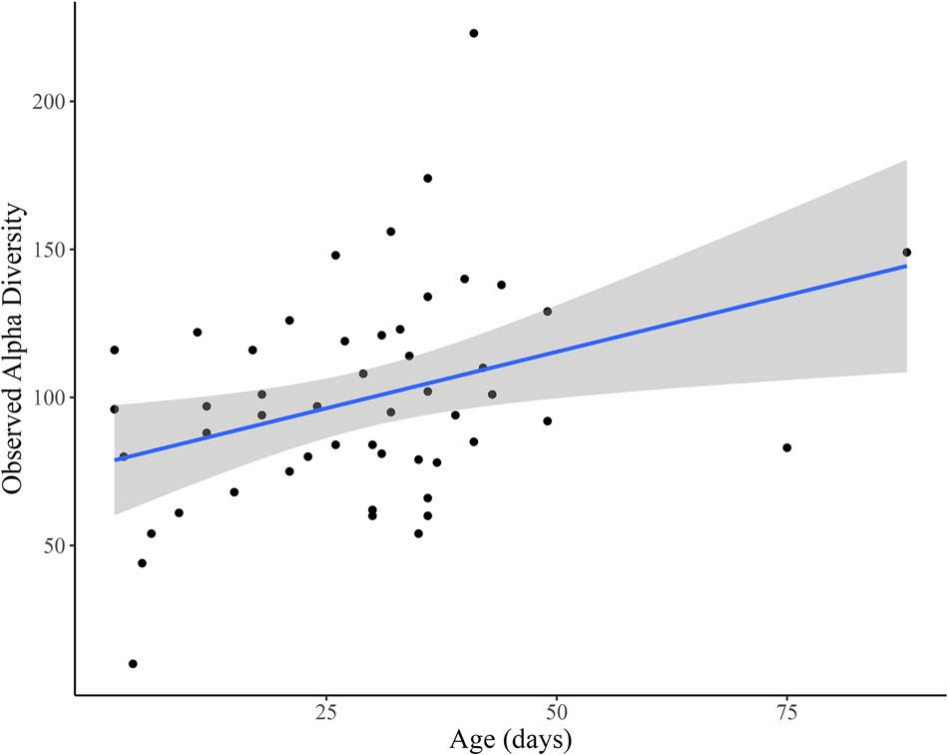
Changes in Observed bacterial alpha diversity in relation to increasing age of juvenile sage-grouse (measured in days). The solid line represents the linear regression line, shaded area represents the 95% confidence interval.

No significant differences in alpha diversity were observed with respect to antibiotic use or management condition (Table S4). Age (measured in days) was positively associated with ASV richness (*β* = 0.69 ± 0.32 *SE*, *t* = 2.17, *P* = 0.04, Fig. 2), but was not a significant variable when assessing Shannon evenness or Simpson diversity (Table S4).

**Figure 3:**
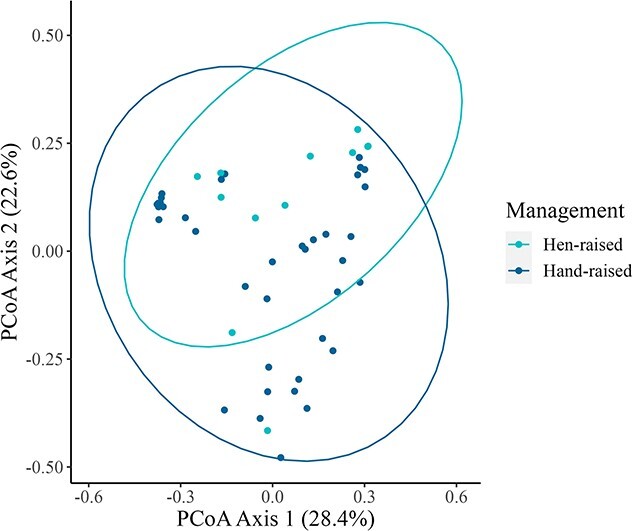
PCoA plot of bacterial community composition based on weighted UniFrac metric of juvenile fecal samples, coloured based on management type. Numbers in parenthesis refer to the variance explained by the ordination axes. Ellipses denote the 95% confidence interval.

**Figure 4:**
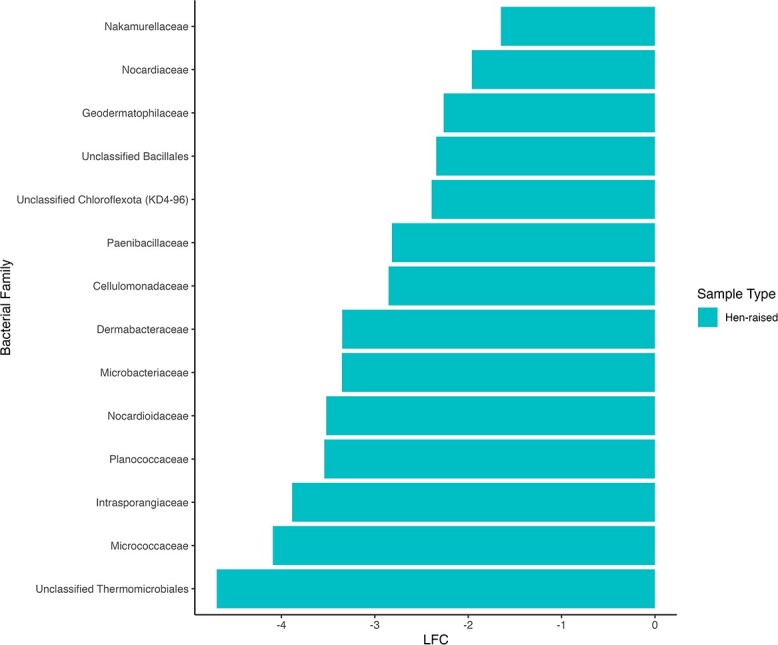
Plot of bacterial families with significantly different abundance in the fecal microbiome of juveniles based on management type. All families were more abundant in samples from hen-raised chicks. LFC = log-fold change in abundance of taxon between groups.

Antibiotic use in juveniles (*n* = 26 not treated, *n* = 24 treated) did not significantly affect microbiome beta diversity (Table S5), but chick age and sample location were significantly associated with Aitchison distance (*P* = 0.02, *P* < 0.01) and unweighted UniFrac distance (*P* = 0.03, *P* < 0.01) measures of beta diversity, but not weighted UniFrac distances (Table S5). However, among pairwise comparisons of Aitchison and unweighted UniFrac distances between sample locations, no pairs of sample locations showed statistically significant differences in beta diversity after Holm–Bonferroni adjustment. Chick management style (*n* = 12 for hen-raised, *n* = 38 for incubated and hand-raised) had a significant effect on all measures of beta diversity (Aitchison, *P* < 0.01; unweighted UniFrac, *P* < 0.01; weighted UniFrac, *P* = 0.03; Fig. 3, Table S5). Post hoc tests of differences in beta dispersion between management styles indicated that hen-raised chicks had more variable microbiomes in Aitchison (difference = 0.225, lower estimate = 0.080, upper estimate = 0.37, *P* = 0.003), but not weighted UniFrac (*P* = 0.50) or unweighted UniFrac (*P* = 0.12) space. Differential abundance analysis revealed 14 bacterial families that were significantly more abundant in hen-raised chicks, and no families that were significantly more abundant in hand-raised chicks (Table S6, Fig. 4).

### Developmental changes in the microbiome of greater sage-grouse

**Figure 5:**
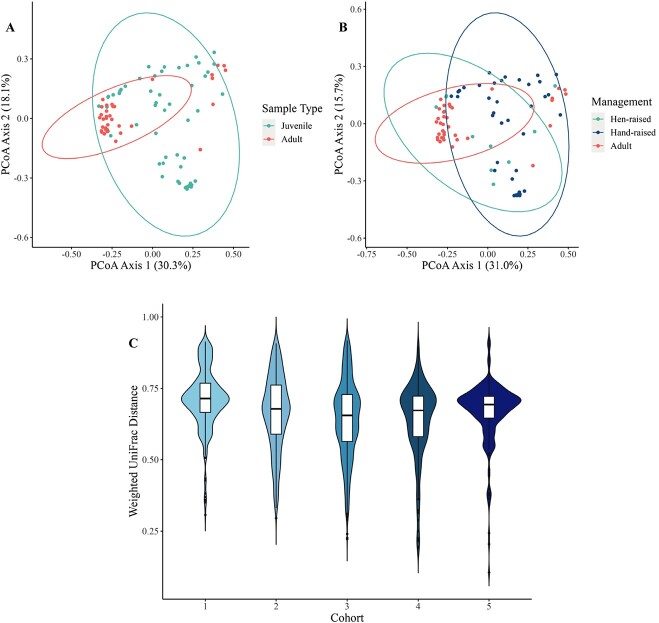
PCoA plot of bacterial community composition based on weighted UniFrac metric of juvenile and adult fecal samples, coloured based on sample type with juveniles combined (A) and coloured based on management type (B). Numbers in parenthesis refer to the variance explained by the ordination axes. Ellipses denote the 95% confidence interval. Violin plot of pairwise weighted UniFrac distance between juvenile and adult fecal samples separated and coloured by age cohort (C). Lower values of distance indicate a more similar bacterial community. Cohort 4 is the only cohort not significantly different from adults.

There were no significant differences in alpha diversity between juvenile and adult samples (Observed *P* = 0.545, Shannon *P* = 0.312, Simpson *P* = 0.259). However, microbiome composition differed between juvenile and adult age classes, based on analyses of unweighted UniFrac, weighted UniFrac and Aitchison distance beta diversity measures (Table 1, Fig. 5a). No difference in Aitchison distance (*P* = 0.39) or weighted UniFrac (*P* = 0.49) beta dispersion were observed between adults and juveniles. However, adults exhibited more variable microbiomes among unweighted UniFrac measures of beta diversity (difference = 0.050, lower estimate = 0.019, upper estimate = 0.073, *P* = 0.001).

**Table 1:** Summary statistics of PERMANOVA comparisons of bacterial beta diversity measures between juvenile sage-grouse fecal samples to adults. Juvenile samples were then separated into management type and compared again to adult samples. *F* = test statistic from PERMANOVA, *Df* = degrees of freedom, *R^2^* = explanatory power and *P* = p value. Bolded p values indicate a statistically significant difference (α < 0.05)

	Aitchison				Unweighted UniFrac				Weighted UniFrac			
	*F*	*Df*	*R^2^*	*P*	*F*	*Df*	*R^2^*	*P*	*F*	*Df*	*R^2^*	*P*
Juveniles vs. adults	10.45	1,89	0.105	**0.0001**	12.21	1,89	0.121	**0.0001**	8.16	1,89	0.084	**0.0001**
Hand-raised vs. adults	12.07	1,71	0.145	**0.0001**	14.08	1,71	0.166	**0.0001**	8.88	1,71	0.111	**0.0001**
Hen-raised vs. adults	2.37	1,45	0.050	**0.016**	2.30	1,45	0.049	**0.024**	1.53	1,45	0.033	0.151

There was a significant difference in all measures of beta diversity based on age cohort, including adults grouped into one cohort (*P* < 0.01, Table 1). Pairwise comparisons between juvenile cohorts revealed significant differences in beta diversity, consistent among all measures, only between cohorts 2 and 5 and between cohorts 3 and 5 (Table S7). Among post hoc tests of multivariate homogeneity of groups dispersions in Weighted UniFrac space, juveniles of cohort five were found to have less variable microbiomes than adults (*P* (adj.) < 0.01), or juveniles from cohorts one (*P* (adj.) < 0.01), two (*P* (adj.) < 0.01) and three (*P* (adj.) < 0.01). Similar patterns were observed with respect to between-cohort dispersion in Aitchison and unweighted UniFrac space (Table S8).

Seven families were differentially abundant between cohorts 2 and 5 (Table S9, Fig. S2), four of which were more abundant in younger chicks (*Erysipelatoclostridiaceae*, *Monoglobaceae*, *Streptococcaceae* and *Bifidobacteriaceae*) and three which were more abundant in older chicks (*Geodermatophilacae*, an unclassified family of order *Victivallales* and an unclassified family from order *Chloroflexales*). Though significant differences in beta diversity were also noted between cohorts 3 and 5, no families were statistically differentially abundant between these age groups.

There was a significant difference between juvenile cohorts 1, 2 and 3 when compared to adults for all three measures of beta diversity (Table S7, Fig. 5c). In comparing juvenile cohort 4 to adults (when sample collection would have occurred in older birds, but before the diet transition or relocation to soft release pens) a significant difference was noted for Aitchison distance (*q* = 0.03), but not unweighted (*q* = 0.06) or weighted UniFrac measures (*q* = 0.17, Fig. 5c). After diet transition and relocation to soft release pens, there was again a significant difference between cohort 5 and adults for all measures of beta diversity (Aitchison: *q* < 0.01, unweighted UniFrac: *q* < 0.01, weighted UniFrac: *q* = 0.01). Differential abundance testing revealed 16 families that were in higher abundance in cohort 1 samples compared to adults (Table S10a), and 13 of those families were also differentially abundant in cohort 5 samples compared to adults (Table S10b).

Adult samples were also compared to juveniles based on management type. When adults were compared to only incubated and hand-raised chicks, assessment of the differences in Aitchison, unweighted and weighted UniFrac distances all increased (Table 1). When adult samples were compared to only hen-raised chicks, Aitchison and unweighted UniFrac distances were still significantly different, but only by a much smaller margin and weighted UniFrac distance was not significantly different (Table 1, Fig. 5b).

Differential abundance analysis revealed 29 bacterial families that had statistically higher abundance in juvenile samples compared to adults (Table S11a, Fig. S3a). When the same comparison was made between only hand-raised chicks (*n* = 38) and adults, the number of families statistically higher in abundance in juvenile samples increased to 33 (Table S11b, Fig. S3b), and when adults were compared to only hen-raised juveniles (*n* = 12), only one family was differentially abundant, *Lactobacillaceae*, which remained more abundant in juveniles than adults (Table S11c).

## Discussion

### Microbiome composition

The composition of the gastrointestinal bacterial microbiome at the phylum level in greater sage-grouse is consistent with previous studies of the avian microbiome in both free-ranging and managed settings, with *Bacillota* as the most abundant phyla, and *Actinomycetota*, *Bacteroidota* and *Pseudomonadota* also being highly abundant (Kohl, 2012; Waite and Taylor, 2014; Hird *et al.*, 2015; Colston and Jackson, 2016; Grond *et al.*, 2018; Liu *et al.*, 2019). Variations in sample collection methodology, DNA extraction techniques, PCR primer selection, sequencing platforms and taxonomic database selection make further assessment between study results challenging.

### Characterization of the microbiome of adult greater sage-grouse

No significant differences were noted in microbiome diversity measurements between male and female adults, likely indicating that sex does not have a significant influence on the microbiome of managed sage-grouse. This is in contrast to findings in other wild avian species, where differences in community composition based on sex were observed in thick billed murres (*Uria lomvia*), as well as three migratory passerine species (Gongora *et al.*, 2021; Yan *et al.*, 2022). Differences in diet and feeding strategies of males and females during either the reproductive season or migration may have a significant impact on the microbiome, as well as sex. The microbiome of wild great bustards (*Otis tarda*) is known to be influenced by both diet and sex, based on sampling of two flocks dependent on different farmed crops over the winter. Great bustards are known as one of the most sexually dimorphic avian species in terms of size difference, which likely leads to differences in food requirements and alterations in the microbiome (Liu *et al.*, 2020). Sage-grouse are sexually dimorphic, with varied investments related to reproduction know to impact survival in the wild; lekking behaviour in males and incubation and chick rearing in females (Boyko *et al.*, 2004; Blomberg *et al*., 2012). Adult sage-grouse in this study were fed a consistent diet, with no difference in access between males and females. The consistency in both diet and environment appears to be a more important contributor to the composition of the microbiome than differences based on sex in this species. Further studies focused on increased sampling during the breeding and nesting period, and from wild animals with access to varied diets could better determine the impact of reproductive behaviours and circulating sex hormones on the microbiome.

### Effect of antibiotic use on the microbiome

Alpha diversity did not vary according to antibiotic use in either adult or juvenile samples in this study. This contrasts with commonly observed reductions in alpha diversity following antibiotic use in other animal species (Dallas and Warne, 2022), including well beyond withdrawal times for the treatments used (Cuccato *et al.*, 2021). The comparisons differed between groups as assessments were made between juvenile samples that had either been treated at any point with antibiotics or had never been treated (though all were hatched from adults with antibiotic exposure), and adults assessed based on treatment in the previous 6 months. Small effects of antibiotic exposure on beta diversity were noted within adult samples only; however, no bacterial families were differentially abundant based on recent antibiotic use. This may indicate that either zoo-housed sage-grouse return to their established microbiome after a disruption caused by antibiotic use, or the effects of any antibiotic use or other associated alterations related to managed care, are long-lasting and generational. Based on the limited availability of untreated samples and the sampling method of this study (fecal samples vs. intestinal contents), no significant conclusions can be made on the impact of antibiotic use on the microbiome in this population, and further study is recommended.

### Characterization of the microbiome of juvenile greater sage-grouse

There were no significant differences in measures of alpha diversity between juveniles based on management strategy (hen-raised versus hand-raised), but management strategy did contribute significantly to bacterial community membership and structure. These differences suggest that incubation or hand-raising of chicks can significantly impact both relative abundance of microbial species and colonization by more distantly related bacteria. This finding also supports conclusions from research in passerines that suggest the avian microbiome is significantly impacted by environmental factors. A study of the avian brood parasite, the brown-headed cowbird (*Molothrus ater*) and other passerine species discovered that environmental variables including physical location and diet had the strongest influence on the gut microbiota (Hird *et al.,* 2014). A cross-foster study of zebra finches (*Taeniopygia guttata*) to society finches (*Lonchura striata domestica*) in a controlled environment also hypothesized that acquisition of bacterial symbionts primarily occurs through indirect maternal transmission in the nest environment. Researchers noted that chicks had microbial communities that converged with the species that raised them, concluding that paternal care is an important factor in the development of the microbiome (Chen *et al.*, 2020). Hand-raised finches also had an overall lower microbial diversity, again emphasizing the contribution of parental care and the nest environment to the establishment of the microbiome (Chen *et al.*, 2020). Differential abundance analysis between the two management groups of sage-grouse revealed 14 bacterial families that were more abundant in hen-raised chicks. These differentially abundant bacterial families include *Micrococcaceae*, which contains the genus *Arthrobacter*, hypothesized to contribute significantly to detoxification of sage in sage-grouse by degrading phenols and catechols to pyruvate (Kohl *et al.*, 2016). These findings support the previous conclusions that the nest environment, location and parental care all contribute significantly to microbiome development.

### Developmental changes in the microbiome of greater sage-grouse

Among juvenile sage-grouse samples, measures of alpha diversity changed over time with increased numbers of ASVs with increasing age (Fig. 2); however, when compared overall there were no significant differences in alpha diversity between juvenile and adult samples. This increase in ASV richness with age is consistent with other avian research, including a study of domestic chickens that also used fecal samples for overall microbiome assessment (Jurburg *et al.*, 2019), a study of house sparrows that assessed both luminal and mucosal intestinal samples (Kohl *et al*., 2019) and research in arctic shorebirds that included the assessment of the embryonic intestinal microbiome and fecal microbiome of chicks (Grond *et al.,* 2017). The lack of significant differences in measures of alpha diversity overall between juvenile and adult sage-grouse is comparable to studies of the crop microbiome in hoatzins (*Opisthocomus hoazin*) (Godoy-Vitorino *et al.,* 2010) and the fecal microbiome of kākāpō (*Strigops habroptilus*) (Waite *et al.,* 2014). However, several studies, for example in the cloacal microbiome in black-legged kittiwakes (*Rissa tridactyla*) (van Dongen *et al.*, 2013) and the fecal microbiome of barn swallows (*Hirundo rustica*) (Kreisinger *et al.*, 2017) and Eurasian kestrels (*Falco tinnunculus*) (Zhou *et al.*, 2020) note an overall difference in alpha diversity between juveniles and adults. The differences in alpha diversity noted in these studies may be related to diet, life-history traits or the variety of ages included in differing studies. Studies in which a significant difference in alpha diversity was observed between adults and juveniles invariably included chicks less than four weeks of age, whereas this study, and the study of hoatzin and kākāpō, included older animals that have begun to converge on a more stable, adult microbiome (Godoy-Vitorino *et al.*, 2010; Waite *et al.*, 2014). Younger chicks have been shown to have significant changes in the microbiome, likely related to cessation of yolk absorption, increased diet diversity and transient colonization of environmental microbes (Kohl, 2012; Teyssier *et al.*, 2018; Videvall *et al.*, 2019; Chen *et al.*, 2020). By also including older chicks with further intestinal and immune system development, a difference in alpha diversity between juvenile and adult sage-grouse may have been obscured.

Previous avian studies have consistently noted changes in community composition over chick development and/or between chicks and adults (Godoy-Vitorino *et al.*, 2010; van Dongen *et al.*, 2013; Kreisinger *et al.*, 2017; Teyssier *et al.*, 2018; Jurburg *et al.*, 2019; Kohl *et al.*, 2019; Videvall *et al.*, 2019; Zhou *et al.*, 2020). In this study, measures of beta diversity were significantly different overall between juveniles and adults. Pairwise comparisons also found significant differences in all measures of beta diversity between juvenile cohort 2 (3–4 weeks) and 5 (>12 weeks) and cohort 3 (5–6 weeks) and 5, as well as significant differences between most juvenile cohorts (1, 2, 3 and 5) and adults. Although there was a significant difference in Aitchison distance between cohort 4 and adults, methods of beta diversity that weigh the phylogenetic relatedness of ASVs (unweighted and weighted UniFrac distances) were not significantly different. This finding suggests that the gastrointestinal microbiome of juveniles between 7 and 12 weeks of age are starting to converge with adults also housed in managed care. This age range coincides with when hand-raised and hen-raised chicks start to live together in larger enclosures, which may also influence this convergence. Differential abundance analysis identified a number of bacterial families that were more abundant in juvenile samples compared to adult samples, which may indicate transient colonization of microbes prior to the development of a stable adult microbiome.

Large compositional changes in the microbiome within the first 2 weeks of development have been discovered in previous studies in a variety of avian species (Grond *et al.*, 2017; Teyssier *et al.*, 2018; Jurburg *et al.*, 2019; Kohl *et al.*, 2019; Videvall *et al.*, 2019). Cohort 1 in this study spans this entire age range and had a small sample size (*n* = 10) with variable richness (range of 10–122 ASVs per sample). Future studies should focus on increased sampling within this age range to better determine microbial community shifts as they relate to development and better inform potential strategies for early microbiome interventions (Ballou *et al.*, 2016; Jurburg *et al.*, 2019).

Assessments of beta dispersion between age cohorts revealed that juveniles in cohort 5 have less variable microbiomes compared younger chicks, likely due to transient colonization of microbial species in younger animals. Interestingly, these older juveniles sampled after both a diet transition to reduce reliance on pelleted feed and moving to soft release pens on native habitats also had less variable microbiomes compared to managed adult sage-grouse. Some researchers have hypothesized that the microbiomes of healthy individuals will have less variation compared to dysbiotic individuals, which would suggest that these management changes support a healthier microbial community composition (Zaneveld *et al.*, 2017).

Chick management had a significant impact on the microbiome of juveniles and was also found to be significant in the comparison of juvenile samples to adults. When incubated and hand-raised chicks were removed from comparisons, there was no longer a significant difference in community structure of juveniles and adults. Differential abundance testing was also assessed and the number of differentially abundant families between groups decreased from 29 to only one, *Lactobacillaceae*. Increased *Lactobacillaceae* in gastrointestinal flora have shown to experimentally increase weight gain in domestic chickens and ducks (*Anas platyrhynchos domestica*) (Angelakis and Raoult, 2010), and increases in abundance of this family in chicks have been noted in other studies of avian development, suggesting a relationship with periods of rapid growth (Waite *et al.*, 2014; Teyssier *et al.*, 2018). Differential abundance testing comparing adults to incubated and hand-raised juveniles noted many bacterial families that were found in higher abundance in chicks. As has been noted in previous studies, the early-life microbiome of chicks appears to be significantly associated with parental care and environmental acquisition of microbiota (Hird *et al.*, 2014; Teyssier *et al.*, 2018; Chen *et al.*, 2020). These differentially abundant families are likely related to the difference in environment and access to adults experienced by incubated and hand-raised juveniles. Increased abundance of some bacterial families may also be related to closer proximity and increased interactions with human caretakers in hand-raised chicks. Humanization of the microbiome is often discussed as a contribution towards microbiome alterations in mammals under human care, related to both the ingestion of more highly cultivated foods, and increased proximity and interactions with humans, but has not yet been assessed in wild avian studies (Clayton *et al.*, 2016; Trevelline and Moeller, 2022).

Further research is needed to assess if the current diet transition and the length of time grouse spend in pens within their native range prior to release is sufficient to overcome any potential long-term impacts of hand-raising and successive generations hatched under human care on the microbiome of managed birds. If managed care has significantly altered the composition of the sage-grouse microbiome, this may correlate with low survival post-release due to a decreased abundance or absence of bacterial species that aid in the digestion and detoxification of their specialist winter diet, sage brush. Studies of another grouse species with a specialist diet, capercaillie (*Tetrao urogallus*), have hypothesized that the significant difference seen between the cecal microbiome of captive and wild birds may be responsible for high post-release mortality (Wienemann *et al.,* 2011), and noted significantly longer small intestines and ceca in wild animals compared to hand-raised birds (Liukkonen-Anttila *et al.,* 2000). These studies indicate the potential impact of management not only to the microbiome itself, but on the surface area and anatomy of the gastrointestinal tract. Many of the grouse included in the current study are the result of several years of breeding under human care; studies using rodent models show that a diet change results in continued alteration of the microbiome over generations and concluded that after several generations a reintroduction of a traditional diet alone was not sufficient for the reestablishment of microbial diversity (Sonnenburg *et al.*, 2016).

### Limitations and suggestions for further research

Although originally 311 samples were collected for DNA extraction and sequencing, only 91 samples were included in the final analyses. Most samples were unusable due to issues with low yields from DNA sequencing. Low yields following DNA extraction specifically from avian feces using commercially available kits are a known issue for microbiome researchers (Eriksson *et al.*, 2017; Edwards *et al.*, 2023). Future studies should consider collecting a larger number of samples than needed for analysis with the expectation that a significant proportion may be lost due to low DNA yields and inadequate sampling depth.

A study published in 2019 measured seasonal change and regional variation in distinct gut regions (crop, ventriculus, duodenum, cecum and colon) of wild sage-grouse specimens collected in Wyoming and concluded that the crop and cecum had distinct microbiomes with limited overlap with samples from the colon (Drovetski *et al.*, 2019). Fecal collection is a well-documented non-invasive approach for assessment of gut microbiome (Grond *et al.*, 2018) and is therefore a useful approach when working with endangered species like sage-grouse. Gallinaceous birds do pass cecum feces, a dark liquid containing cecum content separate from other digesta, approximately once a day (Fenna and Boag, 1974). These samples have been used in previous studies as a proxy for cecal content without invasive sampling (Wienemann *et al.*, 2011) and would likely be beneficial in studies of the sage-grouse microbiome. Due to requirements for limited disturbance to the managed population, fecal samples were used in this study. Though more accurate than cloacal swabbing (Videvall *et al.*, 2018), samples from feces are likely not thoroughly representative of the relative diversity and richness of the bacterial community throughout length of the gastrointestinal tract.

The 16S rRNA gene amplicon data collected in this research was used only for taxonomic identification of ASVs, future research should focus on sampling from wild individuals and functional metagenomic sequencing to better understand the consequences of an altered microbiome composition under managed care. A previous study utilized metagenomic sequencing on samples from three sage-grouse from Idaho and hypothesized bacteria in the genus *Arthrobacter* are the main source of genes responsible for degrading toxic phenols and catechols from sage brush in the cecum (Kohl *et al.*, 2016). However, this genus was not sequenced in the cecal samples from Wyoming sage-grouse (Drovetski *et al.*, 2019), suggesting there may be other species involved in the detoxification of sage brush, these species may vary geographically over the native range of sage-grouse, or genus-level identification of these species is limited using 16S data and the SILVA database. If the bacterial species involved in sage detoxification is identified within the wild Canadian population, this finding could help guide treatment and manipulation of the microbiome in managed care with the goal of increasing sage-grouse survival post-release.

## Supplementary Material

Web_Material_coae052

## Data Availability

Raw sequence reads used in analysis are publicly available in the Sequence Read Archive of the National Center for Biotechnology Information, under BioProject ID PRJNA1129547.

## References

[ref1] Aldridge CL , Brigham RM (2003) Distribution, abundance, and status of the greater sage-grouse, *Centrocercus urophasianus*, in Canada. Can Field Nat 117: 25–34. 10.5962/p.353854.

[ref2] Alfano N , Courtiol A, Vielgrader H, Timms P, Roca AL, Greenwood AD (2015) Variation in koala microbiomes within and between individuals: effect of body region and captivity status. Sci Rep-UK 5: 10189. 10.1038/srep10189. PMC4426690 25960327

[ref3] Anderson MJ (2017) Permutational multivariate analysis of variance (PERMANOVA). In N Balakrishnan, T Colton, B Everitt, W Piegorsch, F Ruggeri, J Teugels, eds, Wiley StatsRef: Statistics Reference Online. Wiley, Hoboken, pp. 1–15

[ref4] Angelakis E , Raoult D (2010) The increase of *lactobacillus* species in the gut flora of newborn broiler chicks and ducks is associated with weight gain. PloS One 5: e10463. 10.1371/journal.pone.0010463. 20454557 PMC2864268

[ref5] Ballou AL , Ali RA, Mendoza MA, Ellis JC, Hassan HM, Croom WJ, Koci MD (2016) Development of the chick microbiome: how early exposure influences future microbial diversity. Front Vet Sci 3: 2. 10.3389/fvets.2016.00002. 26835461 PMC4718982

[ref6] Black SR , Whiteside DP, Pastor A, Vaasjo E (2019) Captive breeding of greater sage-grouse (*Centrocercus urophasianus*) for reintroduction: Challenges and successes 2014-2019. In Proceedings of the 68th Annual Wildlife Disease Association International Conference, Tahoe City, California, pp. 1–15

[ref7] Blomberg EJ , Sedinger JS, Nonne DV, Atamian MT (2013) Seasonal reproductive costs contribute to reduced survival of female greater sage-grouse. J Avian Biol 44: 149–158. 10.1111/j.1600-048X.2012.00013.x.

[ref8] Boyko AR , Gibson RM, Lucas JR, Brown JS (2004) How predation risk affects the temporal dynamics of avian leks: greater sage grouse versus golden eagles. Am Nat 163: 154–165. 10.1086/380419. 14767844

[ref9] Callahan BJ , McMurdie PJ, Rosen MJ, Han AW, Johnson AJA, Holmes SP (2016) DADA2: high-resolution sample inference from Illumina amplicon data. Nat Methods 13: 581–583. 10.1038/nmeth.3869. 27214047 PMC4927377

[ref10] Caporaso JG , Lauber CL, Walters WA, Berg-Lyons D, Huntley J, Fierer N, Owens SM, Betley J, Fraser L, Bauer M et al. (2012) Ultra-high-throughput microbial community analysis on the Illumina HiSeq and MiSeq platforms. ISME J 6: 1621–1624. 10.1038/ismej.2012.8. 22402401 PMC3400413

[ref11] Chen CY , Chen CK, Chen YY, Fang A, Shaw GT, Hung CM, Wang D (2020) Maternal gut microbes shape the early-life assembly of gut microbiota in passerine chicks via nests. Microbiome 8: 129. 10.1186/s40168-020-00896-9. 32917256 PMC7488855

[ref12] Clayton JB , Vangay P, Huang H, Ward T, Hillmann BM, Al-Ghalith GA, Travis DA, Long HT, Tuan BV, Minh VV et al. (2016) Captivity humanizes the primate microbiome. Proc Natl Acad Sci U S A 113: 10376–10381. 10.1073/pnas.1521835113. 27573830 PMC5027417

[ref13] Colston TJ , Jackson CR (2016) Microbiome evolution along divergent branches of the vertebrate tree of life: what is known and unknown. Mol Ecol 25: 3776–3800. 10.1111/mec.13730. 27297628

[ref14] Connelly JW , Schroeder MA, Sands AR, Braun CE (2000) Guidelines to manage sage-grouse populations and their habitats. Wildlife Soc B 28: 967–985.

[ref15] Cuccato M , Rubiola S, Giannuzzi D, Grego E, Pregel P, Divari S, Cannizzo FT (2021) 16s rRNA sequencing analysis of the gut microbiota in broiler chickens prophylactically administered with antimicrobial agents. Antibiotics (Basel) 10: 146. 10.3390/antibiotics10020146. 33540533 PMC7912790

[ref16] Dahlhausen KE , Doroud L, Firl AJ, Polkinghorne A, Eisen JA (2018) Characterization of shifts of koala (*Phascolarctos cinereus*) intestinal microbial communities associated with antibiotic treatment. PeerJ 6: e4452. 10.7717/peerj.4452. 29576947 PMC5853612

[ref17] Dallas JW , Warne RW (2023) Captivity and animal microbiomes: potential roles of microbiota for influencing animal conservation. Microb Ecol 85: 820–838. 10.1007/s00248-022-01991-0. 35316343

[ref18] Delport TC , Power ML, Harcourt RG, Webster KN, Tetu SG (2016) Colony location and captivity influence the gut microbial community composition of the Australian sea lion (*Neophoca cinerea*). Appl Environ Microbiol 82: 3440–3449. 10.1128/AEM.00192-16. 27037116 PMC4959163

[ref19] Dixon P (2003) Vegan, a package of r functions for community ecology. J Vegetation Science 14: 927–930. 10.1111/j.1654-1103.2003.tb02228.x.

[ref20] van Dongen WFD , White J, Brandl HB, Moodley Y, Merkling T, Leclaire S, Blanchard P, Danchin É, Hatch SA, Wagner RH (2013) Age-related differences in the cloacal microbiota of a wild bird species. BMC Ecol 13: 11. 10.1186/1472-6785-13-11. 23531085 PMC3668179

[ref21] Drovetski SV , O’Mahoney MJV, Matterson KO, Schmidt BK, Graves GR (2019) Distinct microbiotas of anatomical gut regions display idiosyncratic seasonal variation in an avian folivore. Animal Microbiome 1: 2. 10.1186/s42523-019-0002-6. 33499946 PMC7803122

[ref22] Edwards J , Hoffbeck C, West AG, Pas A, Taylor MW (2023) 16s rRNA gene-based microbiota profiles from diverse avian faeces are largely independent of DNA preservation and extraction method. Front Microbiol 14: 1239167. 10.3389/fmicb.2023.1239167. 37675430 PMC10477782

[ref23] Eigeland KA , Lanyon JM, Trott DJ, Ouwerkerk D, Blanshard W, Milinovich GJ, Gulino LM, Martinez E, Merson S, Klieve AV (2012) Bacterial community structure in the hindgut of wild and captive dugongs (*Dugong dugon*). Aquat Mamm 38: 402–411. 10.1578/AM.38.4.2012.402.

[ref24] Eriksson P , Mourkas E, Gonzalez-Acuna D, Olsen B, Ellstrom P (2017) Evaluation and optimization of microbial DNA extraction from fecal samples of wild antarctic bird species. Infect Ecol Epidemiol 7: 1386536. 10.1080/20008686.2017.1386536. 29152162 PMC5678435

[ref25] Fenna L , Boag DA (1974) Filling and emptying of the galliform caecum. Can J Zool 52: 537–540. 10.1139/z74-067. 4832971

[ref26] Flechas SV , Blasco-Zúñiga A, Merino-Viteri A, Ramírez-Castañeda V, Rivera M, Amézquita A (2017) The effect of captivity on the skin microbial symbionts in three *Atelopus* species from the lowlands of Colombia and Ecuador. PeerJ 5: e3594. 10.7717/peerj.3594. 28785515 PMC5541920

[ref27] Godoy-Vitorino F , Goldfarb KC, Brodie EL, Garcia-Amado MA, Michelangeli F, Dominguez-Bello MG (2010) Developmental microbial ecology of the crop of the folivorous hoatzin. ISME J 4: 611–620. 10.1038/ismej.2009.147. 20130656

[ref28] Gongora E , Elliott KH, Whyte L (2021) Gut microbiome is affected by inter-sexual and inter-seasonal variation in diet for thick-billed murres (*Uria lomvia*). Sci Rep 11: 1200. 10.1038/s41598-020-80557-x. 33441848 PMC7806582

[ref29] Grond K , Lanctot RB, Jumpponen A, Sandercock BK (2017) Recruitment and establishment of the gut microbiome in arctic shorebirds. FEMS Microbiol Ecol 93: fix142. 10.1093/femsec/fix142. 29069418

[ref30] Grond K , Sandercock BK, Jumpponen A, Zeglin LH (2018) The avian gut microbiota: community, physiology and function in wild birds. J Avian Biol 49: e01788. 10.1111/jav.01788.

[ref31] Guo W , Mishra S, Wang C, Zhang H, Ning R, Kong F, Zeng B, Zhao J, Li Y (2019) Comparative study of gut microbiota in wild and captive giant pandas (*Ailuropoda melanoleuca*). Genes 10: 827. 10.3390/genes10100827. 31635158 PMC6826394

[ref32] Heinrichs JA , McKinnon DT, Aldridge CL, Moehrenschlager A (2019) Optimizing the use of endangered species in multi-population collection, captive breeding and release programs. Glob Ecol Conserv 17: e00558. 10.1016/j.gecco.2019.e00558.

[ref33] Hernández-Gómez O , Briggler JT, Williams RN (2019) Captivity-induced changes in the skin microbial communities of hellbenders (*Cryptobranchus alleganiensis*). Microb Ecol 77: 782–793. 10.1007/s00248-018-1258-1. 30209587

[ref34] Hird SM , Carstens BC, Cardiff SW, Dittmann DL, Brumfield RT (2014) Sampling locality is more detectable than taxonomy or ecology in the gut microbiota of the brood-parasitic brown-headed cowbird (*Molothrus ater*). PeerJ 2: e321. 10.7717/peerj.321. 24711971 PMC3970801

[ref35] Hird SM , Sanchez C, Carstens BC, Brumfield RT (2015) Comparative gut microbiota of 59 neotropical bird species. Front Microbiol 6: 1403. 10.3389/fmicb.2015.01403. 26733954 PMC4685052

[ref36] Holm S (1979) A simple sequentially rejective multiple test procedure. Scand J Stat 6: 65–70.

[ref37] Jia T , Zhao S, Knott K, Li X, Liu Y, Li Y, Chen Y, Yang M, Lu Y, Wu J et al. (2018) The gastrointestinal tract microbiota of northern white-cheeked gibbons (*Nomascus leucogenys*) varies with age and captive condition. Sci Rep-UK 8: 3214–3214. 10.1038/s41598-018-21117-2. PMC5816653 29453448

[ref38] Jurburg SD , Brouwer MSM, Ceccarelli D, van der Goot J, Jansman AJM, Bossers A (2019) Patterns of community assembly in the developing chicken microbiome reveal rapid primary succession. Microbiology 8: e00821. 10.1002/mbo3.821. PMC6741130 30828985

[ref39] Kelly BJ , Gross R, Bittinger K, Sherrill-Mix S, Lewis JD, Collman RG, Bushman FD, Li H (2015) Power and sample-size estimation for microbiome studies using pairwise distances and PERMANOVA. Bioinformatics 31: 2461–2468. 10.1093/bioinformatics/btv183. 25819674 PMC4514928

[ref40] Kohl KD (2012) Diversity and function of the avian gut microbiota. J Comp Physiol B 182: 591–602. 10.1007/s00360-012-0645-z. 22246239

[ref41] Kohl KD , Brun A, Caviedes-Vidal E, Karasov WH (2019) Age-related changes in the gut microbiota of wild house sparrow nestlings. Ibis 161: 184–191. 10.1111/ibi.12618.

[ref42] Kohl KD , Connelly JW, Dearing MD, Forbey JS (2016) Microbial detoxification in the gut of a specialist avian herbivore, the greater sage-grouse. FEMS Microbiol Lett 363: fnw144. 10.1093/femsle/fnw144. 27242374

[ref43] Kozich JJ , Westcott SL, Baxter NT, Highlander SK, Schloss PD (2013) Development of a dual-index sequencing strategy and curation pipeline for analyzing amplicon sequence data on the MiSeq Illumina sequencing platform. Appl Environ Microbiol 79: 5112–5120. 10.1128/AEM.01043-13. 23793624 PMC3753973

[ref44] Kreisinger J , Kropackova L, Petrzelkova A, Adamkova M, Tomasek O, Martin JF, Michalkova R, Albrecht T (2017) Temporal stability and the effect of transgenerational transfer on fecal microbiota structure in a long distance migratory bird. Front Microbiol 8: 50. 10.3389/fmicb.2017.00050. 28220109 PMC5292904

[ref45] Kuznetsova A , Brockhoff PB, Christensen RHB (2017) LmerTest package: tests in linear mixed effects models. J Stat Softw 82: 1–26. 10.18637/jss.v082.i13.

[ref46] Lan Y , Wang Q, Cole JR, Rosen GL (2012) Using the RDP classifier to predict taxonomic novelty and reduce the search space for finding novel organisms. PloS One 7: e32491. 10.1371/journal.pone.0032491. 22403664 PMC3293824

[ref47] Lin H , Peddada SD (2020) Analysis of compositions of microbiomes with bias correction. Nat Commun 11: 3514. 10.1038/s41467-020-17041-7. 32665548 PMC7360769

[ref48] Liu G , Meng D, Gong M, Li H, Wen W, Wang Y, Zhou J (2020) Effects of sex and diet on gut microbiota of farmland-dependent wintering birds. Front Microbiol 11: 587873. 10.3389/fmicb.2020.587873. 33262746 PMC7688461

[ref49] Liu H , Chen Z, Gao G, Sun C, Li Y, Zhu Y (2019) Characterization and comparison of gut microbiomes in nine species of parrots in captivity. Symbiosis 78: 241–250. 10.1007/s13199-019-00613-7.

[ref50] Liukkonen-Anttila T , Saartoala R, Hissa R (2000) Impact of hand-rearing on morphology and physiology of the capercaillie (*Tetrao urogallus*). Comp Biochem Physiol A Mol Integr Physiol 125: 211–221. 10.1016/s1095-6433(99)00174-9. 10825693

[ref51] Martin M (2011) Cutadapt removes adapter sequences from high-throughput sequencing reads. EMBnetjournal 17: 10–12. 10.14806/ej.17.1.200.

[ref52] Martinez Arbizu P (2017) PairwiseAdonis: pairwise multilevel comparison using Adonis. R package version 04 10.

[ref53] McKenzie VJ , Song SJ, Delsuc F, Prest TL, Oliverio AM, Korpita TM, Alexiev A, Amato KR, Metcalf JL, Kowalewski M et al. (2017) The effects of captivity on the mammalian gut microbiome. Integr Comp Biol 57: 690–704. 10.1093/icb/icx090. 28985326 PMC5978021

[ref54] McMurdie PJ , Holmes S (2013) Phyloseq: an R package for reproducible interactive analysis and graphics of microbiome census data. PloS One 8: e61217. 10.1371/journal.pone.0061217. 23630581 PMC3632530

[ref55] Metcalf JL , Song SJ, Morton JT, Weiss S, Seguin-Orlando A, Joly F, Feh C, Taberlet P, Coissac E, Amir A et al. (2017) Evaluating the impact of domestication and captivity on the horse gut microbiome. Sci Rep 7: 15497. 10.1038/s41598-017-15375-9. 29138485 PMC5686199

[ref56] Nearing JT , Douglas GM, Hayes MG, MacDonald J, Desai DK, Allward N, Jones CMA, Wright RJ, Dhanani AS, Comeau AM et al. (2022) Microbiome differential abundance methods produce different results across 38 datasets. Nat Commun 13: 342. 10.1038/s41467-022-28034-z. 35039521 PMC8763921

[ref57] Pastor AR , Black SR, Whiteside DP, Li Y, Goldsmith DA (2021) Multi-species Clostridial outbreaks in neonatal, juvenile, and adult greater sage-grouse (Centrocercus urophasianus) in a conservation breeding program. In Proceedings of the 69th Annual Wildlife Disease Association International Conference, Cuenca, Spain

[ref58] Petersen C , Round JL (2014) Defining dysbiosis and its influence on host immunity and disease. Cell Microbiol 16: 1024–1033. 10.1111/cmi.12308. 24798552 PMC4143175

[ref59] Quast C , Pruesse E, Yilmaz P, Gerken J, Schweer T, Yarza P, Peplies J, Glockner FO (2013) The SILVA ribosomal RNA gene database project: improved data processing and web-based tools. Nucleic Acids Res 41: D590–D596. 10.1093/nar/gks1219. 23193283 PMC3531112

[ref60] R Core Team (2021) R: A language and environment for statistical computing. R Foundation for Statistical Computing, Vienna, Austria

[ref61] Schroeder MA , Aldridge CL, Apa AD, Bohne JR, Braun CE, Bunnell SD, Connelly JW, Deibert PA, Gardner SC, Hilliard MA et al. (2004) Distribution of sage-grouse in North America. The Condor 106: 363–376. 10.1093/condor/106.2.363.

[ref62] Sheneman L , Evans J, Foster JA (2006) Clearcut: a fast implementation of relaxed neighbor joining. Bioinformatics 22: 2823–2824. 10.1093/bioinformatics/btl478. 16982706

[ref63] Simpson EH (1949) Measurement of diversity. Nature 163: 688. 10.1038/163688a0.

[ref64] Sonnenburg ED , Smits SA, Tikhonov M, Higginbottom SK, Wingreen NS, Sonnenburg JL (2016) Diet-induced extinctions in the gut microbiota compound over generations. Nature 529: 212–215. 10.1038/nature16504. 26762459 PMC4850918

[ref65] Spellerberg IF , Fedor PJ (2003) A tribute to Claude Shannon (1916–2001) and a plea for more rigorous use of species richness, species diversity and the ‘Shannon–wiener’ index. Glob Ecol Biogeogr 12: 177–179. 10.1046/j.1466-822X.2003.00015.x.

[ref66] Sun F , Chen J, Liu K, Tang M, Yang Y (2022) The avian gut microbiota: diversity, influencing factors, and future directions. Front Microbiol 13: 934272. 10.3389/fmicb.2022.934272. 35992664 PMC9389168

[ref67] Teyssier A , Lens L, Matthysen E, White J (2018) Dynamics of gut microbiota diversity during the early development of an avian host: evidence from a cross-foster experiment. Front Microbiol 9: 1524. 10.3389/fmicb.2018.01524. 30038608 PMC6046450

[ref68] Thompson LR , Sanders JG, McDonald D, Amir A, Ladau J, Locey KJ, Prill RJ, Tripathi A, Gibbons SM, Ackermann G et al. (2017) A communal catalogue reveals earth's multiscale microbial diversity. Nature 551: 457–463. 10.1038/nature24621. 29088705 PMC6192678

[ref69] Trevelline BK , Moeller AH (2022) Robustness of mammalian gut microbiota to humanization in captivity. Front Ecol Evol 9: 785089. 10.3389/fevo.2021.785089. 35291481 PMC8920477

[ref70] Videvall E , Song SJ, Bensch HM, Strandh M, Engelbrecht A, Serfontein N, Hellgren O, Olivier A, Cloete S, Knight R et al. (2019) Major shifts in gut microbiota during development and its relationship to growth in ostriches. Mol Ecol 28: 2653–2667. 10.1111/mec.15087. 30916826

[ref71] Videvall E , Strandh M, Engelbrecht A, Cloete S, Cornwallis CK (2018) Measuring the gut microbiome in birds: comparison of faecal and cloacal sampling. Mol Ecol Resour 18: 424–434. 10.1111/1755-0998.12744. 29205893

[ref72] Vlčková K , Gomez A, Petrželková KJ, Whittier CA, Todd AF, Yeoman CJ, Nelson KE, Wilson BA, Stumpf RM, Modrý D et al. (2016) Effect of antibiotic treatment on the gastrointestinal microbiome of free-ranging western lowland gorillas (*Gorilla gorilla gorilla*). Microb Ecol 72: 943–954. 10.1007/s00248-016-0745-5. 26984253

[ref73] Waite DW , Eason DK, Taylor MW (2014) Influence of hand rearing and bird age on the fecal microbiota of the critically endangered kakapo. Appl Environ Microbiol 80: 4650–4658. 10.1128/AEM.00975-14. 24837385 PMC4148800

[ref74] Waite DW , Taylor MW (2014) Characterizing the avian gut microbiota: membership, driving influences, and potential function. Front Microbiol 5: 223. 10.3389/fmicb.2014.00223. 24904538 PMC4032936

[ref75] Wang Q , Garrity GM, Tiedje JM, Cole JR (2007) Naive bayesian classifier for rapid assignment of rRNA sequences into the new bacterial taxonomy. Appl Environ Microbiol 73: 5261–5267. 10.1128/AEM.00062-07. 17586664 PMC1950982

[ref76] West AG , Waite DW, Deines P, Bourne DG, Digby A, McKenzie VJ, Taylor MW (2019) The microbiome in threatened species conservation. Biol Conserv 229: 85–98. 10.1016/j.biocon.2018.11.016.

[ref77] Wickham H , Navarro D, Pedersen TL (2016) ggplot2: Elegant graphics for data analysis. Springer, New York

[ref78] Wienemann T , Schmitt-Wagner D, Meuser K, Segelbacher G, Schink B, Brune A, Berthold P (2011) The bacterial microbiota in the ceca of capercaillie (*Tetrao urogallus*) differs between wild and captive birds. Syst Appl Microbiol 34: 542–551. 10.1016/j.syapm.2011.06.003. 21889862

[ref79] Yan R , Lu M, Zhang L, Yao J, Li S, Jiang Y (2022) Effect of sex on the gut microbiota characteristics of passerine migratory birds. Front Microbiol 13: 917373. 10.3389/fmicb.2022.917373. 36118231 PMC9478027

[ref80] Yilmaz P , Parfrey LW, Yarza P, Gerken J, Pruesse E, Quast C, Schweer T, Peplies J, Ludwig W, Glockner FO (2014) The SILVA and "all-species living tree project (LTP)" taxonomic frameworks. Nucleic Acids Res 42: D643–D648. 10.1093/nar/gkt1209. 24293649 PMC3965112

[ref81] Zaneveld JR , McMinds R, Vega Thurber R (2017) Stress and stability: applying the Anna Karenina principle to animal microbiomes. Nat Microbiol 2: 17121. 10.1038/nmicrobiol.2017.121. 28836573

[ref82] Zhou L , Huo X, Liu B, Wu H, Feng J (2020) Comparative analysis of the gut microbial communities of the Eurasian kestrel (*Falco tinnunculus*) at different developmental stages. Front Microbiol 11: 592539. 10.3389/fmicb.2020.592539. 33391209 PMC7775371

