# Supplementary material for: The impact of management on the fecal microbiome of endangered greater sage-grouse (Centrocercus urophasianus) in a zoo-based conservation program
Source: Conserv Physiol. 2024 Aug 7;12(1):coae052. doi: 10.1093/conphys/coae052 (PMC11304599; doi:10.1093/conphys/coae052)
Supplement: Web_Material_coae052 [file web_material_coae052.pdf]

## SUPPLEMENTARY TABLES

**Supplementary Table 1.** Metadata of all samples used in analyses collected from the managed flock at the Wilder Institute/Calgary Zoo.

| Sample_IDs | Type | Date | Antibiotic[1] | Last dose[2] | Days abx[3] | Drug | DOB | Location[4] | Age (days) | Cohort[5] | Individual. Group[6] | Manage[7] | Sex |
|---|---|---|---|---|---|---|---|---|---|---|---|---|---|
| **109813_1** | Adult | 2020-06-09 | 0 | 2019-09-06 | 277 | Lincomycin | 2017-06-16 | D | 1060 | 6 | I | NA | F |
| **109827_1** | Adult | 2020-06-14 | 0 | 2019-09-06 | 282 | Lincomycin | 2017-06-28 | G | 1060 | 6 | I | NA | M |
| **109780_2_2** | Adult | 2020-06-22 | 0 | 2019-09-06 | 290 | Lincomycin | 2017-06-09 | D | 1095 | 6 | I | NA | F |
| **109827_2** | Adult | 2020-06-23 | 0 | 2019-09-06 | 291 | Lincomycin | 2017-06-28 | G | 1060 | 6 | I | NA | M |
| **109760_2** | Adult | 2020-06-25 | 0 | 2019-09-06 | 293 | Lincomycin | 2017-06-05 | D | 1095 | 6 | I | NA | F |
| **109760_3** | Adult | 2020-06-30 | 0 | 2019-09-06 | 298 | Lincomycin | 2017-06-05 | D | 1095 | 6 | I | NA | F |
| **109578_3** | Adult | 2020-06-30 | 0 | 2019-09-06 | 298 | Lincomycin | 2016-05-26 | G | 1490 | 6 | I | NA | F |
| **109780_3** | Adult | 2020-07-07 | 0 | 2019-09-06 | 305 | Lincomycin | 2017-06-09 | B | 1095 | 6 | I | NA | F |
| **109764_2** | Adult | 2020-07-07 | 0 | 2019-09-06 | 305 | Lincomycin | 2017-06-05 | B | 1125 | 6 | I | NA | F |
| **109813_3** | Adult | 2020-07-10 | 0 | 2019-09-06 | 308 | Lincomycin | 2017-06-16 | D | 1095 | 6 | I | NA | F |
| **109764_3** | Adult | 2020-07-16 | 0 | 2019-09-06 | 314 | Lincomycin | 2017-06-05 | B | 1125 | 6 | I | NA | F |
| **109778_3** | Adult | 2020-07-16 | 0 | 2019-09-06 | 314 | Lincomycin | 2017-06-09 | D | 1125 | 6 | I | NA | F |
| **109813_4** | Adult | 2020-07-16 | 0 | 2019-09-06 | 314 | Lincomycin | 2017-06-16 | B | 1125 | 6 | I | NA | F |
| **109780_4** | Adult | 2020-07-18 | 0 | 2019-09-06 | 316 | Lincomycin | 2017-06-09 | B | 1125 | 6 | I | NA | F |
| **109799_3** | Adult | 2020-07-31 | 0 | 2019-09-06 | 329 | Lincomycin | 2017-06-13 | D | 1125 | 6 | I | NA | M |
| **109823_2** | Adult | 2020-07-31 | 0 | 2019-09-06 | 329 | Lincomycin | 2017-06-26 | D | 1125 | 6 | I | NA | M |
| **109957_1** | Adult | 2020-07-31 | 1 | 2019-04-20 | 468 | Enrofloxacin | 2018-06-01 | B | 760 | 6 | I | NA | F |
| **110458_4** | Adult | 2020-08-11 | 1 | 2020-07-26 | 16 | Lincomycin | 2019-06-01 | B | 425 | 6 | I | NA | F |
| **110028_1** | Adult | 2020-08-12 | 0 | 2019-09-06 | 341 | Lincomycin | 2018-06-17 | D | 760 | 6 | I | NA | M |
| **109957_2** | Adult | 2020-08-12 | 1 | 2019-04-20 | 480 | Enrofloxacin | 2018-06-01 | D | 790 | 6 | I | NA | F |
| **110323_6** | Adult | 2020-08-25 | 0 | 2019-08-27 | 364 | Lincomycin | 2019-05-07 | D | 455 | 6 | I | NA | M |
| **109823_3** | Adult | 2020-08-25 | 0 | 2019-09-06 | 354 | Lincomycin | 2017-06-26 | D | 1125 | 6 | I | NA | M |
| **110028_2** | Adult | 2020-08-26 | 0 | 2019-09-06 | 355 | Lincomycin | 2018-06-17 | G | 790 | 6 | I | NA | M |
| **109764_4** | Adult | 2020-08-26 | 0 | 2019-09-06 | 355 | Lincomycin | 2017-06-05 | G | 1155 | 6 | I | NA | F |

| | | | | | | | | | | | | | |
|---|---|---|---|---|---|---|---|---|---|---|---|---|---|
| **109799_5** | Adult | 2020-08-26 | 0 | 2019-09-06 | 355 | Lincomycin | 2017-06-13 | D | 1155 | 6 | I | NA | M |
| **109812_2** | Adult | 2020-08-27 | 0 | 2019-09-06 | 356 | Lincomycin | 2017-05-16 | D | 1185 | 6 | I | NA | M |
| **109436_4** | Adult | 2020-09-24 | 1 | 2020-08-24 | 31 | TMS | 2015-05-04 | G | 1945 | 6 | I | NA | M |
| **109972_2** | Adult | 2020-12-06 | 1 | 2020-09-11 | 86 | Lincomycin | 2018-06-06 | S | 910 | 6 | I | NA | M |
| **109723_2** | Adult | 2020-12-09 | 1 | 2020-09-11 | 89 | Lincomycin | 2017-05-20 | B | 1275 | 6 | I | NA | M |
| **109766_5** | Adult | 2020-12-11 | 1 | 2020-09-11 | 91 | Lincomycin | 2017-06-05 | S | 1275 | 6 | I | NA | M |
| **109780_7** | Adult | 2020-12-11 | 1 | 2020-09-15 | 87 | TMS | 2017-06-09 | S | 1275 | 6 | I | NA | F |
| **109540_5** | Adult | 2021-01-06 | 1 | 2020-09-15 | 113 | TMS | 2016-05-03 | S | 1700 | 6 | I | NA | F |
| **109780_8** | Adult | 2021-01-09 | 1 | 2020-09-15 | 116 | TMS | 2017-06-09 | S | 1305 | 6 | I | NA | F |
| **109972_4** | Adult | 2021-02-09 | 1 | 2020-09-11 | 151 | Lincomycin | 2018-06-06 | S | 970 | 6 | I | NA | M |
| **110532_2** | Adult | 2021-02-12 | 1 | 2020-09-11 | 154 | Lincomycin | 2019-06-18 | S | 575 | 6 | I | NA | F |
| **110817_1** | Juvenile | 2020-06-03 | 0 | NA | NA | NA | 2020-05-29 | M | 5 | 1 | I | 1 | NA |
| **110813_1** | Juvenile | 2020-06-04 | 0 | NA | NA | NA | 2020-05-24 | M | 11 | 1 | I | 1 | NA |
| **110814_1** | Juvenile | 2020-06-08 | 0 | NA | NA | NA | 2020-05-24 | M | 15 | 2 | I | 1 | NA |
| **110964_1** | Juvenile | 2020-06-16 | 1 | 2020-06-16 | 0 | Enrofloxacin | 2020-06-12 | C | 4 | 1 | I | 1 | NA |
| **110979_1** | Juvenile | 2020-06-17 | 0 | NA | NA | NA | 2020-06-15 | M | 2 | 1 | I | 1 | NA |
| **110921_1** | Juvenile | 2020-06-17 | 1 | 2020-06-17 | 0 | Enrofloxacin | 2020-06-08 | M | 9 | 1 | I | 1 | NA |
| **110967_1** | Juvenile | 2020-06-19 | 0 | NA | NA | NA | 2020-06-13 | M | 6 | 1 | I | 1 | NA |
| **110813_2** | Juvenile | 2020-06-19 | 1 | 2020-06-15 | 4 | Enrofloxacin | 2020-05-24 | C | 26 | 2 | I | 1 | NA |
| **110872_1** | Juvenile | 2020-06-25 | 0 | NA | NA | NA | 2020-06-04 | C | 21 | 2 | I | 1 | NA |
| **110912_2** | Juvenile | 2020-06-25 | 1 | 2020-06-15 | 10 | Enrofloxacin | 2020-06-07 | C | 18 | 2 | I | 1 | NA |
| **110814_3** | Juvenile | 2020-06-25 | 1 | 2020-06-19 | 6 | Enrofloxacin, Metronidazole | 2020-05-24 | C | 32 | 3 | I | 1 | NA |
| **111015_1** | Juvenile | 2020-06-27 | 1 | 2020-06-27 | 0 | TMS | 2020-06-25 | M | 2 | 1 | I | 1 | NA |
| **111016_1** | Juvenile | 2020-06-28 | 1 | 2020-06-28 | 0 | TMS | 2020-06-25 | M | 3 | 1 | I | 1 | NA |
| **110923_2** | Juvenile | 2020-06-30 | 0 | NA | NA | NA | 2020-06-09 | C | 21 | 2 | I | 1 | NA |
| **110911_2** | Juvenile | 2020-06-30 | 0 | NA | NA | NA | 2020-06-07 | C | 23 | 2 | I | 1 | NA |
| **110872_2** | Juvenile | 2020-06-30 | 0 | NA | NA | NA | 2020-06-04 | C | 26 | 2 | I | 1 | NA |
| **G110871_4** | Juvenile | 2020-06-30 | 0 | NA | NA | NA | 2020-06-03 | B | 27 | 2 | G | 0 | NA |

| | | | | | | | | | | | | | |
|---|---|---|---|---|---|---|---|---|---|---|---|---|---|
| **110967_3** | Juvenile | 2020-06-30 | 1 | 2020-06-27 | 3 | TMS | 2020-06-13 | C | 17 | 2 | I | 1 | NA |
| **110964_3** | Juvenile | 2020-06-30 | 1 | 2020-06-27 | 3 | TMS | 2020-06-12 | C | 18 | 2 | I | 1 | NA |
| **G110905_5** | Juvenile | 2020-07-01 | 0 | NA | NA | NA | 2020-06-07 | D | 24 | 2 | G | 0 | NA |
| **110817_4** | Juvenile | 2020-07-02 | 0 | NA | NA | NA | 2020-05-29 | C | 34 | 3 | I | 1 | NA |
| **110908_3** | Juvenile | 2020-07-07 | 0 | NA | NA | NA | 2020-06-07 | C | 30 | 3 | I | 1 | NA |
| **110911_3** | Juvenile | 2020-07-07 | 0 | NA | NA | NA | 2020-06-07 | C | 30 | 3 | I | 1 | NA |
| **G110900_5** | Juvenile | 2020-07-07 | 0 | NA | NA | NA | 2020-06-06 | B | 31 | 3 | G | 0 | NA |
| **G110880_6** | Juvenile | 2020-07-07 | 0 | NA | NA | NA | 2020-06-05 | B | 32 | 3 | G | 0 | NA |
| **110872_3** | Juvenile | 2020-07-07 | 0 | NA | NA | NA | 2020-06-04 | C | 33 | 3 | I | 1 | NA |
| **111015_3** | Juvenile | 2020-07-07 | 1 | 2020-07-01 | 6 | TMS | 2020-06-25 | C | 12 | 1 | I | 1 | NA |
| **111017_2** | Juvenile | 2020-07-07 | 1 | 2020-07-01 | 6 | TMS | 2020-06-25 | C | 12 | 1 | I | 1 | NA |
| **110912_4** | Juvenile | 2020-07-07 | 1 | 2020-06-15 | 22 | Enrofloxacin | 2020-06-07 | C | 30 | 3 | I | 1 | NA |
| **110889_5** | Juvenile | 2020-07-07 | 1 | 2020-06-17 | 20 | Enrofloxacin | 2020-06-06 | C | 31 | 3 | I | 1 | NA |
| **110814_4** | Juvenile | 2020-07-07 | 1 | 2020-06-19 | 18 | Enrofloxacin, Metronidazole | 2020-05-24 | C | 44 | 4 | I | 1 | NA |
| **110878_1** | Juvenile | 2020-07-10 | 0 | NA | NA | NA | 2020-06-05 | C | 35 | 3 | I | 0 | NA |
| **110870_1** | Juvenile | 2020-07-10 | 0 | NA | NA | NA | 2020-06-03 | C | 37 | 3 | I | 0 | NA |
| **110813_5** | Juvenile | 2020-07-12 | 1 | 2020-06-15 | 27 | Enrofloxacin | 2020-05-24 | C | 49 | 4 | I | 1 | NA |
| **110814_5** | Juvenile | 2020-07-12 | 1 | 2020-06-19 | 23 | Enrofloxacin, Metronidazole | 2020-05-24 | C | 49 | 4 | I | 1 | NA |
| **110908_4** | Juvenile | 2020-07-16 | 0 | NA | NA | NA | 2020-06-07 | C | 39 | 3 | I | 1 | NA |
| **G110880_7** | Juvenile | 2020-07-16 | 0 | NA | NA | NA | 2020-06-05 | B | 41 | 3 | G | 0 | NA |
| **G110871_6** | Juvenile | 2020-07-16 | 0 | NA | NA | NA | 2020-06-03 | G | 43 | 4 | G | 0 | NA |
| **110952_5** | Juvenile | 2020-07-16 | 1 | 2020-06-27 | 19 | TMS | 2020-06-11 | C | 35 | 3 | I | 1 | NA |
| **110959_1** | Juvenile | 2020-07-17 | 0 | NA | NA | NA | 2020-06-11 | C | 36 | 3 | I | 0 | NA |
| **110961_1** | Juvenile | 2020-07-17 | 0 | NA | NA | NA | 2020-06-11 | C | 36 | 3 | I | 0 | NA |
| **110902_1** | Juvenile | 2020-07-17 | 0 | NA | NA | NA | 2020-06-07 | C | 40 | 3 | I | 0 | NA |
| **110964_6** | Juvenile | 2020-07-23 | 1 | 2020-06-27 | 26 | TMS | 2020-06-12 | C | 41 | 3 | I | 1 | NA |
| **110952_6** | Juvenile | 2020-07-23 | 1 | 2020-06-27 | 26 | TMS | 2020-06-11 | C | 42 | 3 | I | 1 | NA |
| **111017_4** | Juvenile | 2020-07-24 | 1 | 2020-07-01 | 23 | TMS | 2020-06-25 | C | 29 | 3 | I | 1 | NA |

| | | | | | | | | | | | | | |
|---|---|---|---|---|---|---|---|---|---|---|---|---|---|
| **111015_6** | Juvenile | 2020-07-31 | 1 | 2020-07-01 | 30 | TMS | 2020-06-25 | C | 36 | 3 | I | 1 | NA |
| **111016_6** | Juvenile | 2020-07-31 | 1 | 2020-07-01 | 30 | TMS | 2020-06-25 | C | 36 | 3 | I | 1 | NA |
| **111017_5** | Juvenile | 2020-07-31 | 1 | 2020-07-01 | 30 | TMS | 2020-06-25 | C | 36 | 3 | I | 1 | NA |
| **110900_2** | Juvenile | 2020-08-20 | 0 | NA | NA | NA | 2020-06-06 | C | 75 | 4 | I | 0 | NA |
| **110814_6** | Juvenile | 2020-08-20 | 1 | 2020-06-19 | 62 | Enrofloxacin, Metronidazole | 2020-05-24 | C | 88 | 4 | I | 1 | NA |
| **ABSR_0909_2** | Juvenile | 2020-09-09 | NA | NA | NA | NA | NA | D | NA | 5 | G | NA | NA |
| **SKSR_0910_1** | Juvenile | 2020-09-10 | NA | NA | NA | NA | NA | D | NA | 5 | G | NA | NA |
| **ABSR_0917_1** | Juvenile | 2020-09-17 | NA | NA | NA | NA | NA | D | NA | 5 | G | NA | NA |
| **SKSR_0917_2** | Juvenile | 2020-09-17 | NA | NA | NA | NA | NA | D | NA | 5 | G | NA | NA |
| **SKSR_1002_2** | Juvenile | 2020-10-02 | NA | NA | NA | NA | NA | D | NA | 5 | G | NA | NA |
| **ABSR_1028_1** | Juvenile | 2020-10-28 | NA | NA | NA | NA | NA | D | NA | 5 | G | NA | NA |

[1]Binary variable for antibiotic use, in adults 0 = no antibiotic use in the previous 6 months, 1 = antibiotic treatment in the previous 6 months
[2]Date of last antibiotic dose, [3]Number of days since last antibiotic dose
[4]Sample collection location, B= wooden bench, C= crate, D= dirt, G= grass, M= mat, S= snow
[5]Age cohort, 1= 1-2 weeks, 2= 3-4 weeks, 3= 5-6 weeks, 4= 7-12 weeks, 5= >12 weeks, 6= adult
[6]Individual or group sample, group samples taken from hen-raised chicks and from oldest juveniles in soft release pens
[7]Juvenile management, 0 = hen-raised, 1= incubated and hen-raised

**Supplementary Figure 1:** Observed bacterial diversity (number of ASVs) rarefaction curve of sage-grouse fecal microbiomes. Each solid line represents an individual sample, the vertical dotted line represents the value at which each sample was rarefied (8112 reads).

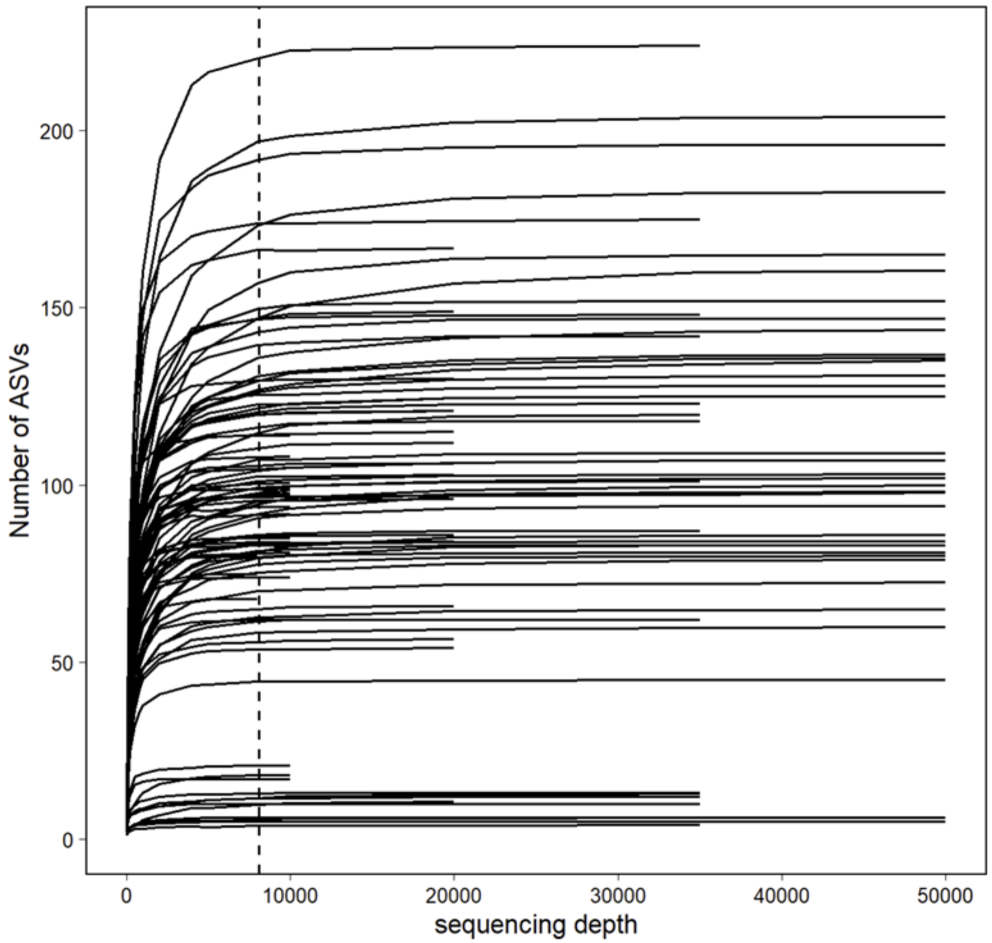

**Supplementary Table 2.** Alpha diversity analysis of adult samples using linear mixed effect models. Sex, antibiotic use, and sample location treated as fixed effects; individual bird identification treated as a random effect. $\beta$ = estimate from linear mixed effects model, *SE* = standard error, *Df* = degrees of freedom, *t*= t value, *Pr*(>|t|) = pseudo p value from mixed effects model.

| Variable | Observed | | | | | Shannon | | | | | Simpson | | | | |
|---|---|---|---|---|---|---|---|---|---|---|---|---|---|---|---|
| | $\beta$ | *SE* | *Df* | *t* | *Pr*(>\|t\|) | $\beta$ | *SE* | *Df* | *t* | *Pr*(>\|t\|) | $\beta$ | *SE* | *Df* | *t* | *Pr*(>\|t\|) |
| **Sex** | -0.089 | 0.114 | 15.265 | -0.782 | 0.446 | -0.164 | 0.568 | 15.623 | -0.289 | 0.777 | -0.089 | 0.114 | 15.265 | -0.782 | 0.446 |
| **Antibiotic Use** | 0.251 | 0.153 | 18.928 | 1.640 | 0.118 | 1.089 | 0.780 | 19.480 | 1.396 | 0.178 | 0.251 | 0.153 | 18.928 | 1.640 | 0.118 |
| **Location (Grass)*** | 0.181 | 0.196 | 24.316 | 0.923 | 0.365 | 0.388 | 1.021 | 24.083 | 0.380 | 0.707 | 0.181 | 0.196 | 24.316 | 0.923 | 0.365 |
| **Location (Wooden Bench)*** | -0.019 | 0.167 | 22.108 | -0.114 | 0.910 | -0.330 | 0.864 | 23.354 | -0.382 | 0.706 | -0.019 | 0.167 | 22.108 | -0.114 | 0.910 |
| **Location (Dirt)*** | 0.218 | 0.189 | 23.860 | 1.157 | 0.259 | 0.620 | 0.978 | 23.244 | 0.634 | 0.532 | 0.218 | 0.189 | 23.860 | 1.157 | 0.259 |

* Sample locations all compared to snow, due to presumed lowest chance of environmental bacterial contamination

**Supplementary Table 3.** Summary statistics of PERMANOVA comparisons of bacterial beta diversity measures of adult samples. *F* = test statistic from PERMANOVA, *Df* = degrees of freedom, $R^2$ = explanatory power, and *P* = p value. Bolded p values indicate a statistically significant difference ($\alpha$ <0.05).

| Variable | Aitchison | | | | Unweighted UniFrac | | | | Weighted UniFrac | | | |
|---|---|---|---|---|---|---|---|---|---|---|---|---|
| | *F* | *Df* | $R^2$ | *P* | *F* | *Df* | $R^2$ | *P* | *F* | *Df* | $R^2$ | *P* |
| **Sex** | 1.919 | 1,28 | 0.049 | 0.061 | 1.394 | 1,28 | 0.037 | 0.188 | 1.151 | 1,28 | 0.030 | 0.306 |
| **Antibiotic Use** | 2.073 | 1,28 | 0.053 | **0.042** | 2.137 | 1,28 | 0.057 | **0.039** | 1.351 | 1,28 | 0.036 | 0.204 |
| **Last Antibiotic Treatment (Days)** | 2.283 | 1,28 | 0.059 | **0.027** | 1.304 | 1,28 | 0.035 | 0.226 | 2.457 | 1,28 | 0.065 | **0.036** |
| **Location** | 1.444 | 3,28 | 0.111 | 0.089 | 1.523 | 3,28 | 0.122 | 0.072 | 1.516 | 3,28 | 0.120 | 0.102 |

**Supplementary Table 4.** Alpha diversity analysis of juvenile samples using linear mixed effect models. Management type, antibiotic use, and age (in days) treated as fixed effects; individual bird identification treated as a random effect. $\beta$ = estimate from linear mixed effects model, *SE* = standard error, *Df* = degrees of freedom, *t*= t value, *Pr*(>|t|) = pseudo p value from mixed effects model. Bolded values indicate statistical significance (α <0.05).

| Variable | Observed | | | | | Shannon | | | | | Simpson | | | | |
|---|---|---|---|---|---|---|---|---|---|---|---|---|---|---|---|
| | $\beta$ | *SE* | *Df* | *t* | *Pr*(>\|t\|) | $\beta$ | *SE* | *Df* | *t* | *Pr*(>\|t\|) | $\beta$ | *SE* | *Df* | *t* | *Pr*(>\|t\|) |
| **Management Type** | -8.289 | 14.974 | 30.349 | -0.554 | 0.584 | 0.200 | 0.273 | 29.025 | 0.734 | 0.469 | 0.056 | 0.052 | 31.505 | 1.077 | 0.290 |
| **Antibiotic Use** | 12.811 | 12.218 | 28.966 | 1.049 | 0.303 | -0.039 | 0.223 | 28.290 | -0.176 | 0.862 | -0.032 | 0.042 | 29.431 | -0.767 | 0.449 |
| **Age (Days)** | 0.688 | 0.318 | 45.163 | 2.165 | **0.036** | 0.011 | 0.006 | 45.841 | 1.947 | 0.058 | 0.002 | 0.001 | 42.972 | 1.445 | 0.156 |

**Supplementary Table 5.** Summary statistics of PERMANOVA comparisons of bacterial beta diversity measures of juvenile samples. *F* = test statistic from PERMANOVA, *Df* = degrees of freedom, $R^2$ = explanatory power, and *P* = p value. Bolded p values indicate a statistically significant difference (α <0.05).

| Variable | Aitchison | | | | Unweighted UniFrac | | | | Weighted UniFrac | | | |
|---|---|---|---|---|---|---|---|---|---|---|---|---|
| | *F* | *Df* | $R^2$ | *P* | *F* | *Df* | $R^2$ | *P* | *F* | *Df* | $R^2$ | *P* |
| **Management Type** | 3.189 | 1,42 | 0.057 | **0.002** | 4.588 | 1,42 | 0.078 | **0.0002** | 2.274 | 1,42 | 0.044 | **0.033** |
| **Antibiotic Use** | 0.882 | 1,42 | 0.016 | 0.537 | 0.956 | 1,42 | 0.016 | 0.446 | 0.577 | 1,42 | 0.011 | 0.814 |
| **Age (days)** | 2.067 | 1,42 | 0.037 | **0.016** | 2.108 | 1,42 | 0.036 | **0.027** | 1.610 | 1,42 | 0.031 | 0.122 |
| **Location** | 1.885 | 4,42 | 0.134 | **0.0003** | 2.096 | 4,42 | 0.143 | **0.001** | 1.366 | 4,42 | 0.106 | 0.082 |

**Supplementary Table 6.** Summary of differential abundance analysis assessment using ANCOM-BC indicating bacterial families that had a statistically significant difference in abundance between hen-raised and hand-raised juveniles. *LFC* = log-fold change in abundance of taxon between groups, negative log fold change indicates increased abundance in hen-raised samples. *SE* = standard error, *W* = test-statistic from ANCOM-BC, and *Q* = q value. The q value is the p value after adjustment using the Holm-Bonferroni method to account for biases from multiple comparisons.

| **Bacterial Family** | ***LFC*** | ***SE*** | ***W*** | ***Q*** |
|---|---|---|---|---|
| Unclassified *Thermomicrobiales* | -4.692 | 0.620 | -7.574 | 2.57E-12 |
| *Micrococcaceae* | -4.092 | 1.064 | -3.846 | 0.0076 |
| *Intrasporangiaceae* | -3.885 | 0.696 | -5.580 | 1.68E-06 |
| *Planococcaceae* | -3.541 | 1.017 | -3.483 | 0.0293 |
| *Nocardioidaceae* | -3.520 | 0.713 | -4.938 | 5.44E-05 |
| *Microbacteriaceae* | -3.352 | 0.711 | -4.715 | 0.0002 |
| *Dermabacteraceae* | -3.349 | 0.714 | -4.690 | 0.0002 |
| *Cellulomonadaceae* | -2.853 | 0.660 | -4.322 | 0.0010 |
| *Paenibacillaceae* | -2.817 | 0.724 | -3.891 | 0.0064 |
| Unclassified *Chloroflexota (*KD4-96*)* | -2.392 | 0.639 | -3.743 | 0.0113 |
| Unclassified *Bacillales* | -2.342 | 0.596 | -3.933 | 0.0055 |
| *Geodermatophilaceae* | -2.263 | 0.623 | -3.633 | 0.0168 |
| *Nocardiaceae* | -1.961 | 0.530 | -3.701 | 0.0131 |
| *Nakamurellaceae* | -1.650 | 0.480 | -3.436 | 0.0342 |

**Supplementary Table 7.** Summary statistics of PERMANOVA, pairwise comparisons of bacterial beta diversity measures between sage-grouse fecal samples based on age. Juvenile samples are separated into age cohorts for pairwise comparisons, cohort 1: 1-2 weeks, cohort 2: 3-4 weeks, cohort 5: 5-6 weeks, cohort 4: 7-12 weeks, and cohort 5: >12 weeks. *F* = test statistic from PERMANOVA, pairwise testing, *Df* = degrees of freedom, $R^2$ = explanatory power, and *Q* = q value. The q value is the p value after adjustment using the Holm-Bonferroni method to account for biases from multiple comparisons. Bolded q values indicate a statistically significant difference (α <0.05).

| Comparison | Aitchison | | | | Unweighted UniFrac | | | | Weighted UniFrac | | | |
|---|---|---|---|---|---|---|---|---|---|---|---|---|
| | *F* | *Df* | $R^2$ | *Q* | *F* | *Df* | $R^2$ | *Q* | *F* | *Df* | $R^2$ | *Q* |
| Cohort 1 vs. 2 | 2.21 | 1, 19 | 0.104 | 0.180 | 2.39 | 1, 19 | 0.112 | 0.138 | 1.60 | 1, 19 | 0.078 | 0.064 |
| Cohort 1 vs. 3 | 1.99 | 1, 31 | 0.060 | 0.168 | 2.36 | 1, 31 | 0.071 | 0.120 | 1.83 | 1, 31 | 0.056 | 0.600 |
| Cohort 1 vs. 4 | 1.24 | 1,14 | 0.081 | 0.168 | 1.68 | 1,14 | 0.107 | 0.436 | 1.11 | 1,14 | 0.073 | 0.912 |
| Cohort 1 vs. 5 | 1.85 | 1,14 | 0.116 | 0.42 | 2.04 | 1,14 | 0.127 | 0.395 | 1.95 | 1,14 | 0.122 | 0.606 |
| Cohort 1 vs. Adults | 5.98 | 1,43 | 0.122 | **0.0015** | 6.15 | 1,43 | 0.125 | **0.002** | 5.20 | 1,43 | 0.108 | **0.003** |
| Cohort 2 vs. 3 | 1.34 | 1,32 | 0.040 | 0.420 | 1.22 | 1,32 | 0.037 | 0.723 | 0.90 | 1,32 | 0.027 | 0.912 |
| Cohort 2 vs. 4 | 2.31 | 1,15 | 0.134 | 0.120 | 2.45 | 1,15 | 0.140 | 0.120 | 1.93 | 1,15 | 0.114 | 0.600 |
| Cohort 2 vs. 5 | 4.80 | 1,15 | 0.242 | **0.020** | 4.99 | 1,15 | 0.249 | **0.002** | 4.95 | 1,15 | 0.248 | **0.026** |
| Cohort 2 vs. Adults | 6.13 | 1,44 | 0.122 | **0.0015** | 6.42 | 1,44 | 0.127 | **0.002** | 4.24 | 1,44 | 0.089 | **0.033** |
| Cohort 3 vs. 4 | 1.46 | 1,27 | 0.051 | 0.420 | 1.21 | 1,27 | 0.043 | 0.723 | 1.51 | 1,27 | 0.053 | 0.640 |
| Cohort 3 vs. 5 | 3.48 | 1,27 | 0.114 | **0.0015** | 3.21 | 1,27 | 0.106 | **0.03** | 3.97 | 1,27 | 0.128 | **0.026** |
| Cohort 3 vs. Adults | 5.68 | 1,56 | 0.920 | **0.002** | 6.15 | 1,56 | 0.099 | **0.002** | 3.81 | 1,56 | 0.064 | **0.033** |
| Cohort 4 vs. 5 | 1.23 | 1,10 | 0.109 | 0.420 | 1.30 | 1,10 | 0.115 | 0.723 | 1.05 | 1,10 | 0.095 | 0.912 |
| Cohort 4 vs. Adults | 3.26 | 1,39 | 0.770 | **0.027** | 2.95 | 1,39 | 0.070 | 0.063 | 2.67 | 1,39 | 0.064 | 0.171 |
| Cohort 5 vs. Adults | 5.96 | 1,39 | 0.132 | **0.0015** | 4.68 | 1,39 | 0.107 | **0.002** | 5.23 | 1,39 | 0.118 | **0.014** |

**Supplementary Table 8.** Summary statistics of Tukey's multiple comparison tests for beta diversity dispersion among different juvenile age cohorts as well as adult samples.

| Comparison | Aitchison | | | | Unweighted UniFrac | | | | Weighted UniFrac | | | |
|---|---|---|---|---|---|---|---|---|---|---|---|---|
| | *Difference* | *Lower* | *Upper* | *P adj.* | *Difference* | *Lower* | *Upper* | *P adj.* | *Difference* | *Lower* | *Upper* | *P adj.* |
| Cohort 1 vs. 2 | 0.167 | -0.579 | 0.914 | 0.986 | -0.024 | -0.112 | 0.063 | 0.965 | 0.006 | -0.107 | 0.118 | 1.000 |
| Cohort 1 vs. 3 | 0.386 | -0.261 | 1.033 | 0.510 | 0.020 | -0.056 | 0.096 | 0.975 | 0.012 | -0.086 | 0.109 | 0.999 |
| Cohort 1 vs. 4 | 0.433 | -0.449 | 1.315 | 0.708 | -0.017 | -0.120 | 0.087 | 0.997 | -0.040 | -0.172 | 0.093 | 0.953 |
| Cohort 1 vs. 5 | -0.567 | -1.449 | 0.315 | 0.425 | -0.080 | -0.184 | 0.023 | 0.222 | -0.196 | -0.328 | -0.063 | **0.001** |
| Cohort 1 vs. Adults | 0.225 | -0.388 | 0.838 | 0.892 | 0.056 | -0.016 | 0.128 | 0.224 | 0.015 | -0.077 | 0.108 | 0.997 |
| Cohort 2 vs. 3 | 0.219 | -0.407 | 0.845 | 0.910 | 0.044 | -0.030 | 0.117 | 0.508 | 0.006 | -0.088 | 0.100 | 1.000 |
| Cohort 2 vs. 4 | 0.266 | -0.601 | 1.133 | 0.947 | 0.008 | -0.094 | 0.109 | 1.000 | -0.045 | -0.176 | 0.085 | 0.912 |
| Cohort 2 vs. 5 | -0.734 | -1.601 | 0.133 | 0.145 | -0.056 | -0.158 | 0.046 | 0.601 | -0.201 | -0.332 | -0.071 | **0.0003** |
| Cohort 2 vs. Adults | 0.058 | -0.533 | 0.648 | 1.000 | 0.080 | 0.011 | 0.149 | **0.014** | 0.009 | -0.080 | 0.098 | 1.000 |
| Cohort 3 vs. 4 | 0.047 | -0.736 | 0.830 | 1.000 | -0.036 | -0.128 | 0.056 | 0.857 | -0.051 | -0.169 | 0.067 | 0.801 |
| Cohort 3 vs. 5 | -0.953 | -1.736 | -0.170 | **0.008** | -0.100 | -0.192 | -0.008 | **0.025** | -0.207 | -0.325 | -0.089 | **0.00003** |
| Cohort 3 vs. Adults | -0.161 | -0.620 | 0.297 | 0.908 | 0.036 | -0.018 | 0.090 | 0.377 | 0.004 | -0.066 | 0.073 | 1.000 |
| Cohort 4 vs. 5 | -1.000 | -1.986 | -0.014 | **0.045** | -0.063 | -0.179 | 0.052 | 0.603 | -0.156 | -0.305 | -0.007 | 0.034 |
| Cohort 4 vs. Adults | -0.208 | -0.963 | 0.547 | 0.966 | 0.073 | -0.016 | 0.161 | 0.173 | 0.055 | -0.059 | 0.169 | 0.723 |
| Cohort 5 vs. Adults | 0.792 | 0.037 | 1.547 | **0.034** | 0.136 | 0.047 | 0.225 | **0.0003** | 0.211 | 0.097 | 0.325 | **0.00001** |

**Supplementary Table 9.** Summary of differential abundance analysis assessment using ANCOM-BC indicating bacterial families that had a statistically significant difference in abundance between juveniles in cohort 2 (3-4 weeks) and juveniles in cohort 5 (>12 weeks). *LFC* = log-fold change in abundance of taxon between groups, positive log fold change indicates increased abundance in cohort 5 samples, negative log fold change indicates increased abundance in cohort 2 samples. *SE* = standard error, *W* = test-statistic from ANCOM-BC, and *Q* = q value. The q value is the p value after adjustment using the Holm-Bonferroni method to account for biases from multiple comparisons.

| **Bacterial Family** | ***LFC*** | ***SE*** | ***W*** | ***Q*** |
|---|---|---|---|---|
| *Geodermatophilaceae* | 3.302 | 0.694 | 4.757 | 0.0001 |
| Unclassified *Chloroflexota* (KD4-96) | 2.696 | 0.805 | 3.349 | 0.048 |
| Unclassified *Victivalles* | 2.681 | 0.804 | 3.335 | 0.049 |
| *Erysipelatoclostridiaceae* | -3.149 | 0.836 | -3.768 | 0.010 |
| *Monoglobaceae* | -4.125 | 1.046 | -3.943 | 0.005 |
| *Streptococcaceae* | -4.769 | 1.199 | -3.976 | 0.004 |
| *Bifidobacteriaceae* | -4.786 | 1.180 | -4.057 | 0.003 |

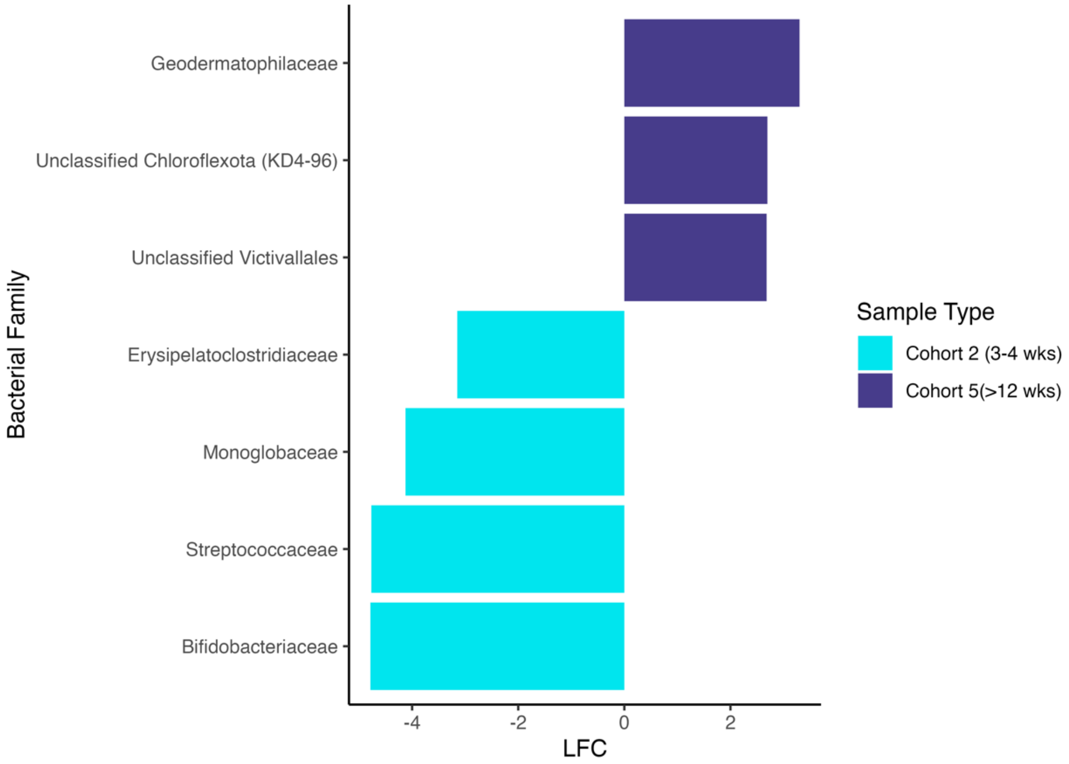


**Figure S2.** Plot of bacterial families with significantly different abundance in the fecal microbiome between juvenile cohorts 2 and 5. LFC = log-fold change in abundance of taxon between groups.

**Supplementary Table 10.** Summary of differential abundance analysis assessment using ANCOM-BC indicating bacterial families that had a statistically significant difference in abundance between juveniles in cohort 1 (1-2 weeks) and adults **(A)**.

| **(A) Bacterial Family** | ***LFC*** | ***SE*** | ***W*** | ***Q*** |
|---|---|---|---|---|
| *Bacteroidaceae* | 5.678 | 0.898 | 6.325 | 2.10E-08 |
| *Oscillospiraceae* | 5.433 | 0.894 | 6.081 | 9.70E-08 |
| *Desulfovibrionaceae* | 5.138 | 0.585 | 8.786 | 1.35E-16 |
| *Lachnospiraceae* | 4.908 | 1.272 | 3.858 | 0.009 |
| *Rikenellaceae* | 4.651 | 1.240 | 3.750 | 0.013 |
| *Barnesiellaceae* | 3.939 | 0.857 | 4.598 | 0.0003 |
| *Butyricicoccaceae* | 3.917 | 0.940 | 4.166 | 0.002 |
| Unclassified *Clostridia* (Vadin BB60) | 3.848 | 0.483 | 7.976 | 1.30E-13 |
| *Coriobacteriaceae* | 3.611 | 0.708 | 5.103 | 2.68E-05 |
| *Marinifilaceae* | 3.546 | 0.546 | 6.493 | 7.08E-09 |
| *Veillonellaceae* | 3.495 | 0.557 | 6.277 | 2.83E-08 |
| *Akkermansiaceae* | 3.210 | 0.863 | 3.719 | 0.015 |
| Unclassified *Oscillospirales* | 3.203 | 0.899 | 3.562 | 0.026 |
| *Tannerellaceae* | 3.154 | 0.410 | 7.689 | 1.26E-12 |
| *Anaerovoracaceae* | 2.579 | 0.662 | 3.896 | 0.007 |
| Unclassified *Victivalles* | 2.473 | 0.516 | 4.794 | 0.0001 |

Summary of differential abundance analysis assessment using ANCOM-BC indicating bacterial families that had a statistically significant difference in abundance between juveniles in cohort 5 (>12 weeks) and adults **(B)**. *LFC* = log-fold change in abundance of taxon between groups, positive log fold change indicates increased abundance in juvenile samples. *SE* = standard error, *W* = test-statistic from ANCOM-BC, and *Q* = q value. The q value is the p value after adjustment using the Holm-Bonferroni method to account for biases from multiple comparisons.

| **(B) Bacterial Family** | ***LFC*** | ***SE*** | ***W*** | ***Q*** |
|---|---|---|---|---|
| *Anaerovoracaceae* | 3.245 | 0.757 | 4.286 | 0.001 |
| *Bacteroidaceae* | 5.239 | 1.118 | 4.687 | 0.0002 |
| *Barnesiellaceae* | 5.637 | 0.942 | 5.984 | 1.74E-07 |
| Unclassified *Clostridia* (Vadin BB60) | 5.412 | 0.650 | 8.331 | 6.82E-15 |
| *Coriobacteriaceae* | 5.282 | 0.688 | 7.681 | 1.33E-12 |
| *Desulfovibrionaceae* | 5.319 | 0.619 | 8.588 | 7.58E-16 |
| *Marinifilaceae* | 5.145 | 0.739 | 6.965 | 2.72E-10 |
| *Oscillospiraceae* | 4.986 | 1.116 | 4.466 | 0.001 |
| Unclassified *Oscillospirales* | 4.025 | 1.036 | 3.887 | 0.008 |
| *Rikenellaceae* | 6.775 | 1.429 | 4.741 | 0.0002 |
| *Tannerellaceae* | 3.843 | 0.661 | 5.813 | 4.84E-07 |
| Unclassified *Victivalles* | 3.924 | 0.585 | 6.707 | 1.63E-09 |
| *Veillonellaceae* | 4.781 | 0.738 | 6.473 | 7.76E-09 |

**Supplementary Table 11.** Summary of differential abundance analysis assessment using ANCOM-BC indicating bacterial families that had a statistically significant difference in abundance between juveniles and adults **(A)**.

| **(A) Bacterial Family** | ***LFC*** | ***SE*** | ***W*** | ***Q*** |
|---|---|---|---|---|
| *Rikenellaceae* | 4.753 | 0.770 | 6.171 | 4.88E-08 |
| *Lactobacillaceae* | 4.398 | 0.745 | 5.906 | 2.34E-07 |
| *Oscillospiraceae* | 4.323 | 0.560 | 7.725 | 9.72E-13 |
| *Lachnospiraceae* | 4.089 | 0.642 | 6.365 | 1.42E-08 |
| *Ruminococcaceae* | 3.895 | 0.647 | 6.016 | 1.25E-07 |
| *Bacteroidaceae* | 3.731 | 0.624 | 5.977 | 1.55E-07 |
| *Desulfovibrionaceae* | 3.683 | 0.490 | 7.512 | 4.95E-12 |
| *Butyricicoccaceae* | 3.545 | 0.530 | 6.687 | 1.73E-09 |
| *Barnesiellaceae* | 3.521 | 0.580 | 6.070 | 9.09E-08 |
| *Coriobacteriaceae* | 3.474 | 0.510 | 6.813 | 7.62E-10 |
| Unclassified *Oscillospirales* | 3.454 | 0.523 | 6.607 | 2.90E-09 |
| Unclassified *Clostridia* (UCG-014) | 3.236 | 0.592 | 5.469 | 2.94E-06 |
| *Anaerovoracaceae* | 3.230 | 0.484 | 6.676 | 1.84E-09 |
| Unclassified *Clostridia* (Vadin BB60) | 3.212 | 0.448 | 7.175 | 6.07E-11 |
| *Veillonellaceae* | 3.022 | 0.449 | 6.736 | 1.27E-09 |
| *Marinifilaceae* | 3.004 | 0.448 | 6.707 | 1.53E-09 |
| *Eggerthellaceae* | 2.988 | 0.498 | 6.002 | 1.35E-07 |
| *Corynebacteriaceae* | 2.479 | 0.577 | 4.299 | 0.001 |
| *Tannerellaceae* | 2.459 | 0.361 | 6.813 | 7.62E-10 |
| *Erysipelatoclostridiaceae* | 2.407 | 0.472 | 5.095 | 2.20E-05 |
| *Coriobacteriales Incertae Sedis* | 2.372 | 0.461 | 5.145 | 1.71E-05 |
| *Christensenellaceae* | 2.275 | 0.556 | 4.092 | 0.003 |
| Unclassified *Victivalles* | 2.271 | 0.301 | 7.540 | 4.03E-12 |
| *Atopobiaceae* | 1.892 | 0.564 | 3.357 | 0.046 |
| *Acidaminococcaceae* | 1.821 | 0.478 | 3.813 | 0.008 |
| *Anaerofustaceae* | 1.813 | 0.310 | 5.852 | 3.21E-07 |
| *Eubacteriaceae* | 1.787 | 0.252 | 7.096 | 1.07E-10 |
| *Hungateiclostridiaceae* | 1.748 | 0.249 | 7.010 | 1.96E-10 |
| *Hydrogenoanaerobacterium* | 1.687 | 0.241 | 7.005 | 2.00E-10 |

Summary of differential abundance analysis assessment using ANCOM-BC indicating bacterial families that had a statistically significant difference in abundance between hand-raised juveniles and adults **(B)**.

| **(B) Bacterial Family** | ***LFC*** | ***SE*** | ***W*** | ***Q*** |
|---|---|---|---|---|
| *Lachnospiraceae* | 4.790 | 0.683 | 7.011 | 1.90E-10 |
| *Oscillospiraceae* | 4.776 | 0.550 | 8.680 | 3.56E-16 |
| *Rikenellaceae* | 4.605 | 0.823 | 5.594 | 1.47E-06 |
| *Lactobacillaceae* | 4.565 | 0.804 | 5.676 | 9.23E-07 |

| | | | | |
|---|---|---|---|---|
| *Ruminococcaceae* | 4.331 | 0.700 | 6.191 | 4.31E-08 |
| *Butyricicoccaceae* | 4.068 | 0.548 | 7.427 | 9.34E-12 |
| *Bacteroidaceae* | 3.937 | 0.658 | 5.982 | 1.54E-07 |
| *Desulfovibrionaceae* | 3.831 | 0.477 | 8.029 | 8.66E-14 |
| Unclassified *Clostridia* (UCG-014) | 3.794 | 0.649 | 5.844 | 3.52E-07 |
| *Anaerovoracaceae* | 3.697 | 0.527 | 7.016 | 1.85E-10 |
| Unclassified *Oscillospirales* | 3.679 | 0.553 | 6.659 | 2.15E-09 |
| *Coriobacteriaceae* | 3.552 | 0.510 | 6.967 | 2.56E-10 |
| *Barnesiellaceae* | 3.406 | 0.590 | 5.777 | 5.19E-07 |
| *Eggerthellaceae* | 3.403 | 0.520 | 6.540 | 4.58E-09 |
| Unclassified *Clostridia* (Vadin BB60) | 3.099 | 0.428 | 7.240 | 3.68E-11 |
| *Corynebacteriaceae* | 3.064 | 0.639 | 4.792 | 0.0001 |
| *Erysipelatoclostridiaceae* | 3.006 | 0.500 | 6.010 | 1.31E-07 |
| *Veillonellaceae* | 2.928 | 0.447 | 6.551 | 4.34E-09 |
| *Marinifilaceae* | 2.894 | 0.439 | 6.586 | 3.48E-09 |
| *Coriobacteriales Incertae Sedis* | 2.799 | 0.504 | 5.557 | 1.78E-06 |
| *Atopobiaceae* | 2.543 | 0.601 | 4.231 | 0.001 |
| *Monoglobaceae* | 2.440 | 0.642 | 3.802 | 0.009 |
| *Tannerellaceae* | 2.387 | 0.365 | 6.541 | 4.58E-09 |
| *Christensenellaceae* | 2.319 | 0.611 | 3.796 | 0.009 |
| *Anaerofustaceae* | 2.201 | 0.340 | 6.480 | 6.69E-09 |
| Unclassified *Clostridia* | 2.193 | 0.584 | 3.756 | 0.010 |
| Unclassified *Victivalles* | 2.132 | 0.270 | 7.905 | 2.32E-13 |
| *Acidaminococcaceae* | 2.002 | 0.504 | 3.973 | 0.004 |
| *Akkermansiaceae* | 1.989 | 0.576 | 3.451 | 0.032 |
| *Peptococcaceae* | 1.886 | 0.258 | 7.299 | 2.41E-11 |
| *Eubacteriaceae* | 1.850 | 0.240 | 7.710 | 1.08E-12 |
| *Hungateiclostridiaceae* | 1.846 | 0.241 | 7.676 | 1.40E-12 |
| *Hydrogenoanaerobacterium* | 1.726 | 0.212 | 8.143 | 3.44E-14 |

Summary of differential abundance analysis assessment using ANCOM-BC indicating bacterial families that had a statistically significant difference in abundance between hen-raised juveniles and adults **(C)**. *LFC* = log-fold change in abundance of taxon between groups, positive log fold change indicates increased abundance in juvenile samples. *SE* = standard error, *W* = test-statistic from ANCOM-BC, and *Q* = q value. The q value is the p value after adjustment using the Holm-Bonferroni method to account for biases from multiple comparisons.

| **(C) Bacterial Family** | ***LFC*** | ***SE*** | ***W*** | ***Q*** |
|---|---|---|---|---|
| *Lactobacillaceae* | 4.244 | 1.086 | 3.908 | 0.008 |

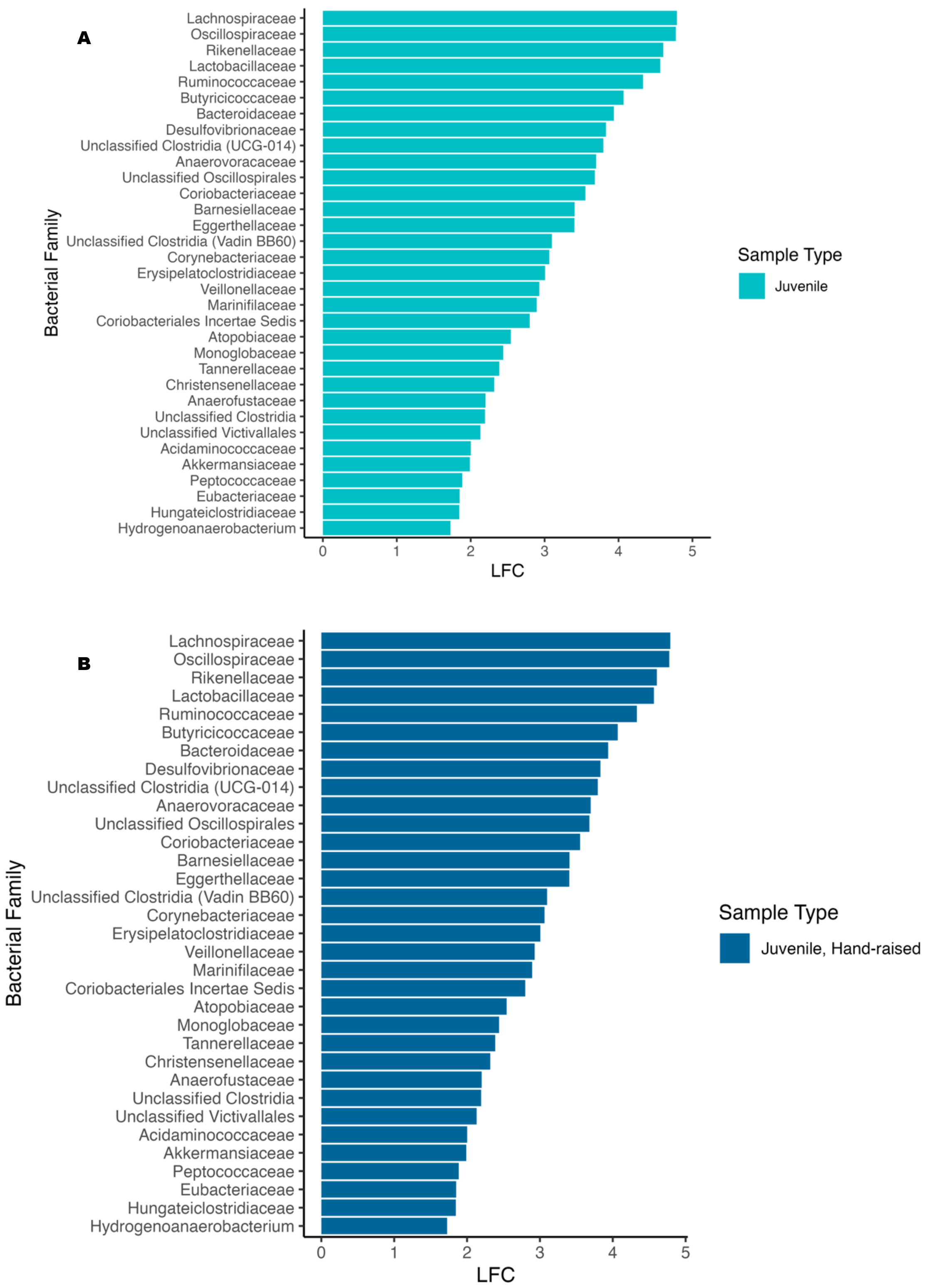


**Figure S3.** Plot of bacterial families with significantly different abundance in the fecal microbiome between juveniles and adults **(A)**, and only hand-raised juveniles and adults **(B)**. LFC = log-fold change in abundance of taxon between groups.

**R script used for statistical analyses:**

```
#Load Packages----
library("ggplot2")
library("microbiome")
library ("phyloseq")
library("devtools")
library("genefilter")
library('plyr')
library("reshape2")
library('vegan')
library('phytools')
library('lmerTest')
library('pairwiseAdonis')
library("ANCOMBC")
library("car")
library("dplyr")
library("readxl")
library("tidyverse")
library("utils")
library("genefilter")
library("ggpubr")
library("Rmisc")

#Load and organize data----
setwd("/Users/emmavaasjo/Grouse R Script")
grouseComm <- read_csv2phyloseq(otu.file = "ASV_table_silva_MSotthart_2022-09-19.csv",
taxonomy.file = "taxonomy_table_silva_MStothart_2022-09-19.csv", metadata.file =
"grouse_metadata_Mstothart_2022-09-19.csv")
#loads csv files to build a phyloseq object (AKA a BIOM file).
#View(sample_data(grouseComm))
Grouse_Phylo_Tree<-read.newick(file="grouseASVFastaAligned.tre")
# Load in ASV phylogenetic tree

grouseMBiom<-merge_phyloseq(grouseComm, Grouse_Phylo_Tree)
#Merge biom file and phylogenetic tree
#OTU file contains the number of reads assigned to each microbial taxon (rows) for each sample that we
sequenced (columns).
#The taxonomy file contains the taxonomic assignments for each microbial taxon in the OTU table (e.g.
Phylum, Class, Order, Family, Genus, Species).
#The metadata is the sample data pertaining to each sampled that we sequenced (date, sex, age etc.).
#The 'read_csv2phyloseq' function takes those input csv files, and binds them into a single phyloseq
object.

# create vector of names of the mitochondrial or chloroplast ASVs/OTUs
chloro_or_mito <- c("ASV_11", "ASV_58", "ASV_281", "ASV_500", "ASV_524", "ASV_528")  #

allTaxa = taxa_names(grouseMBiom) # get a vector off all the taxon names in your samples.
keepTaxa <- allTaxa[!(allTaxa %in% chloro_or_mito)] # get vector of non-chloroplast and non-
mitochondrial names.
#function to create a new phyloseq object with the mitochondrial and chloroplast reads removed
```

```
grouseBiom = prune_taxa(keepTaxa, grouseMBiom)


# Remove Samples that aren't being used.
grouseBiom_Data <- prune_samples(sample_data(grouseBiom)$Type.1 != "Gitract", grouseBiom)
#Remove GI samples
grouseBiom_Data <- prune_samples(sample_data(grouseBiom_Data)$Type.1 != "Wild",
grouseBiom_Data)
#Remove wild samples
grouseBiom_Data <- prune_samples(sample_data(grouseBiom_Data)$Type.1 != "Drovetski",
grouseBiom_Data)
#Remove available sequences from previously published grouse paper

set.seed(123)
grouseBiom_Data_R <- rarefy_even_depth(grouseBiom_Data, rngseed = TRUE)

sample_data(grouseBiom_Data)$Observed <- estimate_richness(grouseBiom_Data_R, measures =
"Observed")[,1]
sample_data(grouseBiom_Data)$Shannon <- estimate_richness(grouseBiom_Data_R, measures =
"Shannon")[,1]
sample_data(grouseBiom_Data)$Simpson <- estimate_richness(grouseBiom_Data_R, measures =
"Simpson")[,1]

# Within Captive Adult Comparisons-----
grouseBiom_Adult <- prune_samples(sample_data(grouseBiom_Adult)$Type.1 != "Juvenile",
grouseBiom_Adult)

sample_data(grouseBiom_Adult)$days_abx <- as.numeric(sample_data(grouseBiom_Adult)$days_abx)
        # Alpha (Simpson, Shannon, Observed)-----
sample_data(grouseBiom_Adult)$Location_S <- factor(sample_data(grouseBiom_Adult)$Location,
levels = c("S", "G", "B", "D"))

#Observed diversity analyses
fm1 <- lmer(scale(sample_data(grouseBiom_Adult)$Observed) ~ sample_data(grouseBiom_Adult)$Sex
+ sample_data(grouseBiom_Adult)$Antibiotic + sample_data(grouseBiom_Adult)$Location_S + (1 |
sample_data(grouseBiom_Adult)$Name))
summary(fm1)

#Shannon Diversity analyses
fm1 <- lmer(sample_data(grouseBiom_Adult)$Shannon ~ sample_data(grouseBiom_Adult)$Sex +
sample_data(grouseBiom_Adult)$Antibiotic + sample_data(grouseBiom_Adult)$Location_S + (1 |
sample_data(grouseBiom_Adult)$Name))
summary(fm1) #Note, these are psueo p-values, because you've run a mixed-effects model

#Simpson Diversity analyses
fm1 <- lmer(sample_data(grouseBiom_Adult)$Simpson ~ sample_data(grouseBiom_Adult)$Sex +
sample_data(grouseBiom_Adult)$Antibiotic + sample_data(grouseBiom_Adult)$Location_S + (1 |
sample_data(grouseBiom_Adult)$Name))
summary(fm1) #Note, these are psueo p-values, because you've run a mixed-effects model

# Beta Diversity (Bray-Curtis, Weighted, and Unweighed UniFrac)-----
```

```
set.seed(123) #create a rarefied dataset for the adult samples
grouseBiom_Adult_R <- rarefy_even_depth(grouseBiom_Adult, rngseed = TRUE, sample.size = 8112)
#here, we can specify the minimum sequencing depth, to allow for consistency across comparisons
grouseBiom_Adult_CLR <- microbiome::transform(grouseBiom_Adult, 'clr')
Grouse.Ordination.CLR.A <- ordinate(grouseBiom_Adult_CLR,
                                    method = "PCoA", distance = "euclidean") #This command will calculate principal
coordinates for the samples, using aitchisons distances

Grouse.Ordination.Wuni.A <- ordinate(grouseBiom_Adult_R,
                                     method = "PCoA", distance = "wunifrac") #This command will calculate principal
coordinates for the samples, using a wunifrac metric

Grouse.Ordination.Uni.A <- ordinate(grouseBiom_Adult_R,
                                    method = "PCoA", distance = "unifrac") #This command will calculate principal
coordinates for the samples, using a unifrac metric

#PERMANOVA tests
grouse.aitch.a<- phyloseq::distance (grouseBiom_Adult_CLR,
                                     "euclidean") #create dissimilarity matrices
grouse.wuni.a <- phyloseq::distance (grouseBiom_Adult_R,
                                     "wunifrac")
grouse.uni.a <- phyloseq::distance (grouseBiom_Adult_R,
                                    "unifrac")

adonis2 (grouse.aitch.a ~ Sex + Antibiotic + Location, as(sample_data (grouseBiom_Adult_R),
"data.frame"), permutations=9999, by = 'margin')

adonis2 (grouse.uni.a ~ Sex + Antibiotic + Location, as(sample_data (grouseBiom_Adult_R),
"data.frame"), permutations=9999, by = 'margin')

adonis2 (grouse.wuni.a ~ Sex + Antibiotic + Location, as(sample_data (grouseBiom_Adult_R),
"data.frame"), permutations=9999, by = 'margin')

#Pairwise adonis to figure out which groups differ based on location
pairwiseAdonis::pairwise.adonis2(grouse.aitch.a ~ Location, as(sample_data(grouseBiom_Adult_R),
"data.frame"), permutations=9999)
p_AitchAd <-c(0.0348, 0.0417, 0.5374, 0.3408, 0.1596, 0.0177, 0.0715, 0.0736, 0.1225, 0.4286)
p.adjust(p_AitchAd, method = "holm") #holm p-value correction
pairwiseAdonis::pairwise.adonis2(grouse.uni.a ~ Location, as(sample_data(grouseBiom_Adult_R),
"data.frame"), permutations=9999) #pairwise adonis to figure out which groups differ
p_UniAd <-c(0.0345, 0.0898, 0.3943, 0.5475, 0.5575, 0.0534, 0.1464, 0.2169, 0.2488, 0.5714)
p.adjust(p_UniAd, method = "holm") #holm p-value correction
pairwiseAdonis::pairwise.adonis2(grouse.wuni.a ~ Location, as(sample_data(grouseBiom_Adult_R),
"data.frame"), permutations=9999) #pairwise adonis to figure out which groups differ
p_WuniAd <-c(0.0773, 0.01428, 0.3519, 0.2312, 0.4393, 0.0078, 0.1372, 0.0588, 0.1307, 0.8571)
p.adjust(p_WuniAd, method = "holm") #holm p-value correction

# Differential Abundance (Adult birds based on “Antibiotic)------
grouseBiom_Adult_Fam <- tax_glom(grouseBiom_Adult, "Family")
```

```
ANCOMoutputA.FDR.Cons <- ancombc2(data = grouseBiom_Adult_Fam, assay_name = "counts",
tax_level = "Family", fix_formula = "Antibiotic", p_adj_method = "holm", group = "Antibiotic", lib_cut
= 1000, struc_zero = FALSE, neg_lb = FALSE, alpha = 0.05, global = TRUE)

results <- ANCOMoutputA.FDR.Cons$res

ancomresults <- as.data.frame(cbind(results$lfc_Antibiotic1, results$se_Antibiotic1,
results$W_Antibiotic1, results$q_Antibiotic1, results$diff_Antibiotic1))
colnames(ancomresults) <- c("LFC", "W", "Q", "SE", "DiffAbund")
ancomresults$Species <- rownames(results)
taxonomy.file <- read.csv("taxonomy_table_silva_MStothart_2022-09-19.csv")
ancomTaxMerge <- merge(ancomresults, taxonomy.file, by.x = "Species", by.y = "X", all.x = TRUE)
write.csv(ancomTaxMerge, "Adult_Antibiotic_ANCOM_Holm_Conservative.csv") #Keep in mind, these
tests are for family level differences

#ancom_results_sig <- ancomTaxMerge[ancomTaxMerge$DiffAbund ==  1]


# Within Juvenile Comparisons----
grouseBiom_Juv <- prune_samples(sample_data(grouseBiom_Juv)$Type.1 != "Adult", grouseBiom_Juv)
grouseBiom_JuvI <- prune_samples(sample_data(grouseBiom_Juv)$Cohort != "5", grouseBiom_Juv) #to
prune soft release

sample_data(grouseBiom_JuvI)$Age_numeric <- as.numeric(sample_data(grouseBiom_JuvI)$Age)
#convert age to numeric data
sample_data(grouseBiom_Juv)$Cohort_numeric <- as.numeric(sample_data(grouseBiom_Juv)$Cohort)

 # Alpha (Simpson, Shannon, Observed)-----
#Observed diversity analyses
fm1 <- lmer(sample_data(grouseBiom_JuvI)$Observed ~ sample_data(grouseBiom_JuvI)$Antibiotic +
sample_data(grouseBiom_JuvI)$Management + sample_data(grouseBiom_JuvI)$Age_numeric + (1 |
sample_data(grouseBiom_JuvI)$Name))

#Shannon Diversity analyses
fm1 <- lmer(sample_data(grouseBiom_JuvI)$Shannon ~ sample_data(grouseBiom_JuvI)$Antibiotic +
sample_data(grouseBiom_JuvI)$Management + sample_data(grouseBiom_JuvI)$Age_numeric + (1 |
sample_data(grouseBiom_JuvI)$Name))
summary(fm1)

#Simpson Diversity analyses
fm1 <- lmer(sample_data(grouseBiom_JuvI)$Simpson ~ sample_data(grouseBiom_JuvI)$Antibiotic +
sample_data(grouseBiom_JuvI)$Management + sample_data(grouseBiom_JuvI)$Age_numeric + (1 |
sample_data(grouseBiom_JuvI)$Name))
summary(fm1)

#Beta Diversity (Bray-Curtis, Weighted, and Unweighed UniFrac)-----
set.seed(123) #create a rarefied dataset for the juvenile samples
grouseBiom_Juv_R <- rarefy_even_depth(grouseBiom_Juv, rngseed = TRUE, sample.size = 8112)
grouseBiom_JuvI_R <- rarefy_even_depth(grouseBiom_JuvI, rngseed = TRUE, sample.size = 8112)

grouseBiom_Juv_CLR <- microbiome::transform(grouseBiom_Juv, 'clr')
```

```
Grouse.Ordination.CLR.J <- ordinate(grouseBiom_Juv_CLR,
                              method = "PCoA", distance = "euclidean") #This command will calculate principal coordinates for the samples, using aitchisons distance
grouseBiom_JuvI_CLR <- microbiome::transform(grouseBiom_JuvI, 'clr')
Grouse.Ordination.CLR.J <- ordinate(grouseBiom_Juv_CLR,
                              method = "PCoA", distance = "euclidean")
Grouse.Ordination.CLR.JI <- ordinate(grouseBiom_JuvI_CLR,
                              method = "PCoA", distance = "euclidean")

Grouse.Ordination.Wuni.J <- ordinate(grouseBiom_Juv_R,
                               method = "PCoA", distance = "wunifrac") #This command will calculate principal coordinates for the samples, using a wunifrac metric

Grouse.Ordination.Wuni.JI <- ordinate(grouseBiom_JuvI_R,
                               method = "PCoA", distance = "wunifrac")

Grouse.Ordination.Uni.J <- ordinate(grouseBiom_Juv_R,
                              method = "PCoA", distance = "unifrac") #This command will calculate principal coordinates for the samples, using a unifrac metric
Grouse.Ordination.Uni.JI <- ordinate(grouseBiom_JuvI_R,
                              method = "PCoA", distance = "unifrac")

#PERMANOVA tests
grouse.aitch.j <- phyloseq::distance (grouseBiom_Juv_CLR,
                               "euclidean") #create dissimilarity matrices
grouse.wuni.j <- phyloseq::distance (grouseBiom_Juv_R,
                               "wunifrac")
grouse.uni.j <- phyloseq::distance (grouseBiom_Juv_R,
                              "unifrac")
grouse.aitch.ji <- phyloseq::distance (grouseBiom_JuvI_CLR,
                                "euclidean")
grouse.wuni.ji <- phyloseq::distance (grouseBiom_JuvI_R,
                               "wunifrac")
grouse.uni.ji <- phyloseq::distance (grouseBiom_JuvI_R,
                              "unifrac")

adonis2 (grouse.aitch.ji ~ Antibiotic + Age_numeric + Management + Location, as(sample_data
(grouseBiom_JuvI_R), "data.frame"), permutations=9999, by = 'margin')

adonis2 (grouse.wuni.ji ~ Antibiotic + Age_numeric + Management + Location, as(sample_data
(grouseBiom_JuvI_R), "data.frame"), permutations=9999, by = 'margin')

adonis2 (grouse.uni.ji ~ Antibiotic + Age_numeric + Management + Location, as(sample_data
(grouseBiom_JuvI_R), "data.frame"), permutations=9999, by = 'margin')

#Pairwise adonis tests to figure out which groups differ based on location
pairwiseAdonis::pairwise.adonis2(grouse.aitch.ji ~ Location, as(sample_data(grouseBiom_JuvI_R),
"data.frame"), permutations=9999)
#B vs C
p_AitchJuv <-c(0.0533, 0.107, 0.1693, 0.5604, 0.1917, 0.05, 0.4885, 0.4, 1)
p.adjust(p_AitchJuv, method = "holm")
```

```
pairwiseAdonis::pairwise.adonis2(grouse.uni.ji ~ Location, as(sample_data(grouseBiom_JuvI_R),
"data.frame"), permutations=9999)
#M vs C
p_UniJuv <-c(0.0202, 0.2247, 0.147, 0.6639, 0.4036, 0.1314, 0.781, 0.2, 0.8)
p.adjust(p_UniJuv, method = "holm")

#Assessment of beta dispersion
uni_dispersion <- betadisper(grouse.uni.ji, sample_data(grouseBiom_JuvI_R)$Management, type =
"centroid", bias.adjust=TRUE, sqrt.dist = TRUE)
TukeyHSD(uni_dispersion)


# Differential Abundance (Juvenile birds based on “Management”)------
grouseBiom_JuvI_Fam <- tax_glom(grouseBiom_JuvI, "Family")

ANCOMoutputJuvI.FDR.Cons <- ancombc2(data = grouseBiom_JuvI_Fam, assay_name = "counts",
tax_level = "Family", fix_formula = "Management", p_adj_method = "holm", group = "Management",
lib_cut = 1000, struc_zero = FALSE, neg_lb = FALSE, alpha = 0.05, global = TRUE)

results <- ANCOMoutputJuvI.FDR.Cons$res

ancomresults <- as.data.frame(cbind(results$lfc_Management1, results$se_Management1,
results$W_Management1, results$q_Management1, results$diff_Management1))

colnames(ancomresults) <- c("LFC", "SE", "W", "Q", "DiffAbund")
ancomresults$Species <- rownames(results)

taxonomy.file <- read.csv("taxonomy_table_silva_MStothart_2022-09-19.csv")
ancomTaxMerge <- merge(ancomresults, taxonomy.file, by.x = "Species", by.y = "X", all.x = TRUE)

write.csv(ancomTaxMerge, "JuvI_Management__ANCOM_Holm_Conservative.csv")
ancom_results_sig <- ancomTaxMerge[ancomTaxMerge$DiffAbund ==  1,] #table of only those that are
differentially abundant

#Differential Abundance (Juvenile birds based on “Cohort” 2 vs. 5)------
grouseBiom_JuvC <- prune_samples(sample_data(grouseBiom_Juv)$Cohort != "1", grouseBiom_Juv)
grouseBiom_JuvC <- prune_samples(sample_data(grouseBiom_JuvC)$Cohort != "3",
grouseBiom_JuvC)
grouseBiom_JuvC <- prune_samples(sample_data(grouseBiom_JuvC)$Cohort != "4",
grouseBiom_JuvC)

grouseBiom_JuvC_Fam <- tax_glom(grouseBiom_JuvC, "Family")

ANCOMoutputJuvC.FDR.Cons <- ancombc2(data = grouseBiom_JuvC_Fam, assay_name = "counts",
tax_level = "Family", fix_formula = "Cohort", p_adj_method = "holm", group = "Cohort", lib_cut =
1000, struc_zero = FALSE, neg_lb = FALSE, alpha = 0.05, global = TRUE)

results <- ANCOMoutputJuvC.FDR.Cons$res
```

```
ancomresults <- as.data.frame(cbind(results$lfc_Cohort5, results$se_Cohort5, results$W_Cohort5,
results$q_Cohort5, results$diff_Cohort5))

colnames(ancomresults) <- c("LFC", "SE", "W", "Q", "DiffAbund")
ancomresults$Species <- rownames(results)
taxonomy.file <- read.csv("taxonomy_table_silva_MStothart_2022-09-19.csv")

ancomTaxMerge <- merge(ancomresults, taxonomy.file, by.x = "Species", by.y = "X", all.x = TRUE)

write.csv(ancomTaxMerge, "JuvI_Cohort25_ANCOM_Holm_Conservative.csv")
ancom_results_sig <- ancomTaxMerge[ancomTaxMerge$DiffAbund ==  1,] #table of only those that are
differentially abundant

# Comparisons of Juvenile versus Adult -----
#Prune soft release samples
grouseBiom_AJI <- prune_samples(sample_data(grouseBiom_AJ)$Cohort != "5", grouseBiom_AJ)

# Alpha (Simpson, Shannon, Observed)-----
#Observed diversity analyses
fm1 <- lmer(sample_data(grouseBiom_AJ)$Observed ~ sample_data(grouseBiom_AJ)$Type.1 + (1 |
sample_data(grouseBiom_AJ)$Name))
summary(fm1)

#Shannon Diversity analyses
fm1 <- lmer(sample_data(grouseBiom_AJ)$Shannon ~ sample_data(grouseBiom_AJ)$Type.1 + (1 |
sample_data(grouseBiom_AJ)$Name))
summary(fm1)

#Simpson Diversity analyses
fm1 <- lmer(sample_data(grouseBiom_AJ)$Simpson ~ sample_data(grouseBiom_AJ)$Type.1 + (1 |
sample_data(grouseBiom_AJ)$Name))
summary(fm1)

# Beta Diversity assessments (Bray-Curtis, Weighted, and Unweighed UniFrac)-----
set.seed(123) #create a rarefied dataset for the adult/juvenile samples
grouseBiom_AJ_R <- rarefy_even_depth(grouseBiom_AJ, rngseed = TRUE, sample.size = 8112) #here,
we can specify the minimum sequencing depth, to allow for consistency across comparisons
grouseBiom_AJI_R <- rarefy_even_depth(grouseBiom_AJI, rngseed = TRUE, sample.size = 8112)

grouseBiom_AJ_CLR <- microbiome::transform(grouseBiom_AJ, 'clr')
Grouse.Ordination.CLR.AJ <- ordinate(grouseBiom_AJ_CLR,
                             method = "PCoA", distance = "euclidean")

grouseBiom_AJI_CLR <- microbiome::transform(grouseBiom_AJI, 'clr')
Grouse.Ordination.CLR.AJI <- ordinate(grouseBiom_AJI_CLR,
                              method = "PCoA", distance = "euclidean")
#This command will calculate principal coordinates for the samples, using aitchisons distances

Grouse.Ordination.Wuni.AJ <- ordinate(grouseBiom_AJ_R,
                              method = "PCoA", distance = "wunifrac")
```

```
Grouse.Ordination.Wuni.AJI <- ordinate(grouseBiom_AJI_R,
                                method = "PCoA", distance = "wunifrac")
#This command will calculate principal coordinates for the samples, using a wunifrac metric

Grouse.Ordination.Uni.AJ <- ordinate(grouseBiom_AJ_R,
                                method = "PCoA", distance = "unifrac")

Grouse.Ordination.Uni.AJI <- ordinate(grouseBiom_AJI_R,
                                method = "PCoA", distance = "unifrac")
#This command will calculate principal coordinates for the samples, using a unifrac metric

#PERMANOVA tests
grouse.aitch.aj <- phyloseq::distance (grouseBiom_AJ_CLR,
                                "euclidean") #create dissimilarity matrices
grouse.wuni.aj <- phyloseq::distance (grouseBiom_AJ_R,
                                "wunifrac")
grouse.uni.aj <- phyloseq::distance (grouseBiom_AJ_R,
                                "unifrac")

adonis2 (grouse.aitch.aj ~ Type.1, as(sample_data (grouseBiom_AJ_R), "data.frame"),
permutations=9999)
adonis2 (grouse.wuni.aj ~ Type.1, as(sample_data (grouseBiom_AJ_R), "data.frame"),
permutations=9999)
adonis2 (grouse.uni.aj ~ Type.1, as(sample_data (grouseBiom_AJ_R), "data.frame"), permutations=9999)

adonis2 (grouse.aitch.aj ~ Cohort, as(sample_data (grouseBiom_AJ_R), "data.frame"),
permutations=9999)
adonis2 (grouse.uni.aj ~ Cohort, as(sample_data (grouseBiom_AJ_R), "data.frame"), permutations=9999)
adonis2 (grouse.wuni.aj ~ Cohort, as(sample_data (grouseBiom_AJ_R), "data.frame"),
permutations=9999)

#Pairwise adonis tests to figure out which groups differ (based on cohort)
pairwiseAdonis::pairwise.adonis2(grouse.aitch.aj ~ Cohort, as(sample_data(grouseBiom_AJ_R),
"data.frame"), permutations=9999)
p_AitchAJ <-c(0.036, 0.026, 0.024, 0.105, 0.0001, 0.138, 0.015, 0.002, 0.0001, 0.105, 0.0001, 0.0002,
0.217, 0.003, 0.0001)
p.adjust(p_AitchAJ, method = "holm")

pairwiseAdonis::pairwise.adonis2(grouse.uni.aj ~ Cohort, as(sample_data(grouseBiom_AJ_R),
"data.frame"), permutations=9999)
p_UniAJ <-c(0.023, 0.015, 0.109, 0.079, 0.0001, 0.241, 0.016, 0.0001, 0.0001, 0.248, 0.003, 0.0001,
0.244, 0.007, 0.0002)
p.adjust(p_UniAJ, method = "holm")

pairwiseAdonis::pairwise.adonis2(grouse.wuni.aj ~ Cohort, as(sample_data(grouseBiom_AJ_R),
"data.frame"), permutations=9999)
p_WuniAJ <-c(0.128, 0.075, 0.304, 0.101, 0.0002, 0.479, 0.079, 0.002, 0.003, 0.141, 0.002, 0.003, 0.313,
0.019, 0.001)
p.adjust(p_WuniAJ, method = "holm")

#Assessment of beta dispersion
```

```
uni_dispersion <- betadisper(grouse.uni.aj, sample_data(grouseBiom_AJ_R)$Cohort, type = "centroid",
bias.adjust=TRUE, sqrt.dist = TRUE)
TukeyHSD(uni_dispersion)

# Differential Abundance (Juvenile versus Adult)------
grouseBiom_AJ_Fam <- tax_glom(grouseBiom_AJ, "Family")

ANCOMoutputAJ.FDR.Cons <- ancombc2(data = grouseBiom_AJ_Fam, assay_name = "counts",
tax_level = "Family", fix_formula = "Type.1", p_adj_method = "holm", group = "Type.1", lib_cut = 1000,
struc_zero = FALSE, neg_lb = FALSE, alpha = 0.05, global = TRUE)

results <- ANCOMoutputAJ.FDR.Cons$res

ancomresults <- as.data.frame(cbind(results$lfc_Type.1Juvenile, results$se_Type.1Juvenile,
results$W_Type.1Juvenile, results$q_Type.1Juvenile, results$diff_Type.1Juvenile))

colnames(ancomresults) <- c("LFC", "SE", "W", "Q", "DiffAbund")
ancomresults$Species <- rownames(results)

taxonomy.file <- read.csv("taxonomy_table_silva_MStothart_2022-09-19.csv")

ancomTaxMerge <- merge(ancomresults, taxonomy.file, by.x = "Species", by.y = "X", all.x = TRUE)

ancom_results_sig <- ancomTaxMerge[ancomTaxMerge$DiffAbund ==  1,] #table of only those that are
differentially abundant

#Differences between juveniles and adults based on management, beta diversity
#comparison on hen-raised to adults
sample_data(grouseBiom_AJI)$ManageHen <- "none"
sample_data(grouseBiom_AJI)$ManageHen[sample_data(grouseBiom_AJI)$Type.1 == "Adult"] <-
"Yes"
sample_data(grouseBiom_AJI)$ManageHen[sample_data(grouseBiom_AJI)$Management == 0] <-
"Yes"

grouseBiom_AJI_M0 <- prune_samples(sample_data(grouseBiom_AJI)$ManageHen != "none",
grouseBiom_AJI)

set.seed(123) #create a rarefied dataset
grouseBiom_AJI_M0_R <- rarefy_even_depth(grouseBiom_AJI_M0, rngseed = TRUE, sample.size =
8112)
grouseBiom_AJI_M0_CLR <- microbiome::transform(grouseBiom_AJI_M0, 'clr')

Grouse.Ordination.CLR.AJIM0 <- ordinate(grouseBiom_AJI_M0_CLR,
                        method = "PCoA", distance = "euclidean")

Grouse.Ordination.Wuni.AJIM0 <- ordinate(grouseBiom_AJI_M0_R,
                        method = "PCoA", distance = "wunifrac")

Grouse.Ordination.Uni.AJM0 <- ordinate(grouseBiom_AJI_M0_R,
                        method = "PCoA", distance = "unifrac")
```

```
grouse.aitch.ajm0 <- phyloseq::distance (grouseBiom_AJI_M0_CLR,
                                  "euclidean") #create dissimilarity matrices
grouse.wuni.ajm0 <- phyloseq::distance (grouseBiom_AJI_M0_R,
                                 "wunifrac")
grouse.uni.ajm0 <- phyloseq::distance (grouseBiom_AJI_M0_R,
                                 "unifrac")

adonis2 (grouse.aitch.ajm0 ~ Type.1, as(sample_data (grouseBiom_AJI_M0_R), "data.frame"),
permutations=9999)
adonis2 (grouse.wuni.ajm0 ~ Type.1, as(sample_data (grouseBiom_AJI_M0_R), "data.frame"),
permutations=9999)
adonis2 (grouse.uni.ajm0 ~ Type.1, as(sample_data (grouseBiom_AJI_M0_R), "data.frame"),
permutations=9999)

#comparison of hand-raised to adults
sample_data(grouseBiom_AJI)$ManageHen2 <- "none"
sample_data(grouseBiom_AJI)$ManageHen2[sample_data(grouseBiom_AJI)$Type.1 == "Adult"] <-
"Yes"
sample_data(grouseBiom_AJI)$ManageHen2[sample_data(grouseBiom_AJI)$Management == 1] <-
"Yes"

grouseBiom_AJI_M1 <- prune_samples(sample_data(grouseBiom_AJI)$ManageHen2 != "none",
grouseBiom_AJI)

set.seed(123)
grouseBiom_AJI_M1_R <- rarefy_even_depth(grouseBiom_AJI_M1, rngseed = TRUE, sample.size =
8112)
grouseBiom_AJI_M1_CLR <- microbiome::transform(grouseBiom_AJI_M1, 'clr')

grouse.aitch.ajm1 <- phyloseq::distance (grouseBiom_AJI_M1_CLR,
                                   "euclidean") #create dissimilarity matrices
grouse.wuni.ajm1 <- phyloseq::distance (grouseBiom_AJI_M1_R,
                                  "wunifrac")
grouse.uni.ajm1 <- phyloseq::distance (grouseBiom_AJI_M1_R,
                                  "unifrac")

adonis2 (grouse.aitch.ajm1 ~ Type.1, as(sample_data (grouseBiom_AJI_M1_R), "data.frame"),
permutations=9999)
adonis2 (grouse.wuni.ajm1 ~ Type.1, as(sample_data (grouseBiom_AJI_M1_R), "data.frame"),
permutations=9999)
adonis2 (grouse.uni.ajm1 ~ Type.1, as(sample_data (grouseBiom_AJI_M1_R), "data.frame"),
permutations=9999)

#differential abundance adult and hen raised
grouseBiom_AJI_M0 <- tax_glom(grouseBiom_AJI_M0, "Family")

ANCOMoutputAJM0.FDR.Cons <- ancombc2(data = grouseBiom_AJI_M0, assay_name = "counts",
tax_level = "Family", fix_formula = "Type.1", p_adj_method = "holm", group = "Type.1", lib_cut = 1000,
struc_zero = FALSE, neg_lb = FALSE, alpha = 0.05, global = TRUE)

results <- ANCOMoutputAJM0.FDR.Cons$res
```

```
ancomresults <- as.data.frame(cbind(results$lfc_Type.1Juvenile, results$se_Type.1Juvenile,
results$W_Type.1Juvenile, results$q_Type.1Juvenile, results$diff_Type.1Juvenile))

colnames(ancomresults) <- c("LFC", "SE", "W", "Q", "DiffAbund")
ancomresults$Species <- rownames(results)
taxonomy.file <- read.csv("taxonomy_table_silva_MStothart_2022-09-19.csv")
ancomTaxMerge <- merge(ancomresults, taxonomy.file, by.x = "Species", by.y = "X", all.x = TRUE)
write.csv(ancomTaxMerge, "Adult_versus_JuvenileHen_ANCOM_Holm_Conservative.csv")
ancom_results_sig <- ancomTaxMerge[ancomTaxMerge$DiffAbund ==  1,] #table of only those that are
differentially abundant

#differential abundance adult and hand raised
grouseBiom_AJI_M1 <- tax_glom(grouseBiom_AJI_M1, "Family")

ANCOMoutputAJM1.FDR.Cons <- ancombc2(data = grouseBiom_AJI_M1, assay_name = "counts",
tax_level = "Family", fix_formula = "Type.1", p_adj_method = "holm", group = "Type.1", lib_cut = 1000,
struc_zero = FALSE, neg_lb = FALSE, alpha = 0.05, global = TRUE)

results <- ANCOMoutputAJM1.FDR.Cons$res

ancomresults <- as.data.frame(cbind(results$lfc_Type.1Juvenile, results$se_Type.1Juvenile,
results$W_Type.1Juvenile, results$q_Type.1Juvenile, results$diff_Type.1Juvenile))

colnames(ancomresults) <- c("LFC", "SE", "W", "Q", "DiffAbund")
ancomresults$Species <- rownames(results)
taxonomy.file <- read.csv("taxonomy_table_silva_MStothart_2022-09-19.csv")
ancomTaxMerge <- merge(ancomresults, taxonomy.file, by.x = "Species", by.y = "X", all.x = TRUE)
write.csv(ancomTaxMerge, "Adult_versus_JuvenileHand_ANCOM_Holm_Conservative.csv")
ancom_results_sig <- ancomTaxMerge[ancomTaxMerge$DiffAbund ==  1,] #table of only those that are
differentially abundant
```
